# Peptides from Different Carcass Elements of Organic and Conventional Pork—Potential Source of Antioxidant Activity

**DOI:** 10.3390/antiox9090835

**Published:** 2020-09-07

**Authors:** Paulina Kęska, Sascha Rohn, Michał Halagarda, Karolina M. Wójciak

**Affiliations:** 1 Department of Animal Raw Materials Technology, University of Life Sciences in Lublin, 20033 Lublin, Poland; paulina.keska@up.lublin.pl; 2 Hamburg School of Food Science, Institute of Food Chemistry, University of Hamburg, 20146 Hamburg, Germany; rohn@chemie.uni-hamburg.de; 3 Department of Food Product Quality, Cracow University of Economics, 31510 Kraków, Poland; michal.halagarda@uek.krakow.pl

**Keywords:** antioxidant peptides, element of pork carcasses, spectrometric analysis

## Abstract

The growing consumer interest in organic foods, as well as, in many cases, the inconclusiveness of the research comparing organic and conventional foods, indicates a need to study this issue further. The aim of the study was to compare the effects of meat origin (conventional vs. organic) and selected elements of the pork carcass (ham, loin, and shoulder) on the meat proteome and the antioxidant potential of its peptides. The peptidomic approach was used, while the ability of antioxidants to scavenge 2,2′-azino-bis-3-ethylbenzthiazoline-6-sulfonic acid (ABTS), to chelate Fe(II) ions, and to reduce Fe(III) was determined. Most peptides were derived from myofibrillary proteins. The meat origin and the element of the pork carcass did not have a significant effect on the proteome. On the other hand, the pork origin and the carcass element significantly affected the iron ion-chelating capacity (Fe(II)) and the reducing power of peptides. In particular, pork ham from conventional rearing systems had the best antioxidant properties in relation to potential antioxidant peptides. This could be a factor for human health, as well as for stabilized meat products (e.g., toward lipid oxidation).

## 1. Introduction

The food consumption pattern has changed over the years. Currently, consumers appreciate foods that, in addition to favorable sensory properties, are characterized by high nutritional value and potential health benefits [1,2]. Therefore, an increasing interest in organic foods can be observed [3,4]. Nonetheless, many consumers still do not believe in the favorable characteristics of organic food products. Moreover, the research findings regarding the extensive, positive effects of the consumption of such foods are not comprehensive enough; in many cases, they are also inconclusive [5,6,7,8], which indicates the need to study this issue further.

Meat consumption, although discussed sometimes quite controversially, is a good source of many highly valuable nutrients [9]. Meat has a complex physical structure and chemical composition, highly sensitive to enzymatic activities. Glycolysis and rigor mortis, occurring in the muscles within the first 24 h of slaughtering, are significant processes that can regulate proteolysis and affect the peptide profile of muscle tissue. Proteins involved in these biochemical processes are subject to complex metabolic regulations that significantly contribute to the final quality of the meat. Moreover, the potential resulting peptides not only have nutritional value, but also carry further benefits for human health [10]. Meat-derived peptides have been shown to have antioxidant, antihypertensive, antidiabetic, antimicrobial, opioid, anticoagulant, and other bioactive effects. Regulation of the immune, gastrointestinal, and neurological responses of these bioactive peptides is an important basis for the prevention of noncommunicable diseases such as hypertension, obesity, diabetes, and other metabolic disorders [11]. In addition to nutritional value, natural antioxidants might also be beneficial for stabilizing products with regard to rancidity via lipid peroxidation [12].

Before being released from parent proteins, peptides remain latent and do not show any bioactive effect. Their potential activity is only activated after the release of specific amino-acid sequences, through gastrointestinal digestion or food processing (e.g., drying, curing, fermentation, or enzymatic hydrolysis). The literature data indicate processed meat products as good sources of peptides with biological activity, particularly dry-cured or fermented meats [13,14,15,16]. For example, the antiradical activity of protein extracts from dry-cured pork loins over a 12-month period of aging was confirmed. Moreover, antiradical activity was further observed in peptic and pancreatic hydrolysates after simulated gastrointestinal digestion [14]. However, the chemical composition of meat also promotes oxidation processes. Ingredients susceptible to oxidation, i.e., polyunsaturated fatty acids, cholesterol, proteins, and pigments, should be in balance with endogenous antioxidant substances. Endogenous antioxidant systems consist of nonenzymatic compounds such as tocopherols, ascorbic acids, carotenoids, and ubiquinols, as well as enzymes (superoxide dismutase, catalase, and glutathione peroxidase). Furthermore, peptides such as anserine and carnosine have high antioxidant activity, acting as free-radical scavengers and metal-ion chelators. Peptide formation via hydrolytic reactions is the main technique used to create antioxidants from proteins, as the resulting peptides have significantly advanced antioxidant potential compared to intact proteins [17,18].

According to the literature, meat peptides can contribute to maintaining the oxidative stability of meat tissue [18,19,20]. Lipids are particularly vulnerable to oxidative factors, especially during meat processing. The carbonyl-based compounds known as secondary lipid oxidation products are characterized by cytotoxic and genotoxic properties [17]. In addition to exogenous/added antioxidants or endogenous small-molecular antioxidants (e.g., vitamins), lipid oxidation in meat and meat products can also be inhibited by proteins as a result of biologically designed mechanisms (such as iron-binding proteins and antioxidant enzymes) or via some non-specific mechanisms. The activity of these mechanisms can be intensified when the conformation of proteins is denatured. Previous studies showed that <3.5 kDa peptides have strong antioxidant activity. They can function as inhibitors of lipid oxidation, leading to color change or discoloration during prolonged storage in uncured roasted beef with acid whey [15,21]. Moreover, the role of proteins, as important components of muscle tissue, in developing the sensory profile of meat products was emphasized. Among them, myofibrillar proteins proved to be particularly good flavor precursors, i.e., active peptides and amino acids. The suppressing (sourness and sweetness) and enhancing (salty and umami) role of peptides was also presented in pork meat based on an in silico approach [22]. Apart from improving the shelf-life and organoleptic qualities of meat, controlling lipid-based oxidative degradation contributes to preventing the negative effects of oxidative stress, which is generally caused by the excessive accumulation of reactive oxygen species (ROS). It is an imbalance between the production and accumulation of ROS and endogenous defense mechanisms (e.g., enzymes, vitamins) in cells and tissues. Consequently, the limited capacity of the biological system to detoxify these reactive products can lead to disease-causing pathological conditions. Importantly, according to some literature reports, elevated levels of oxidative stress play a dominant role in initiating many cardiovascular diseases, diabetes mellitus, and other metabolic disorders [23]. Antioxidants are effective in combating the effects of oxidative stress. In this context, digestive amino acids, peptides, and proteins from different food sources can also act as antioxidants to protect cells and organisms from oxidative damage [17,18].

The composition of oxidation-promoting factors and antioxidants may vary between the meat of different animal species [24,25] or gender [26]. In addition, the animal’s diet plays a significant role in modifying the concentration of antioxidants or pro-oxidative factors [27,28,29,30]. Therefore, different biomolecular compositions of meat from slaughter animals can affect the functionality and use of a particular muscle tissue for specific applications. Thus, it is necessary to know and understand the differences in the biomolecule profiles between different carcass elements of the animal muscles. The understanding of any differences or similarities between them, determined based on the abundance in protein composition and related peptides, can be helpful in achieving this goal. Moreover, the antioxidant potential of peptides from selected elements of organic vs. conventional pork carcasses has not been compared before.

Therefore, the aim of the present study was to investigate the impact of organic and conventional rearing systems on protein stability and peptide formation with regard to their antioxidant activity in pork meat available for consumers. Furthermore, various pork tissues such as ham, loin, and shoulder, in relation to their potential to be used as starting materials for obtaining high-quality functional ingredients or meat products, were characterized.

## 2. Materials and Methods

### 2.1. Meat Samples

The research was conducted on organic and conventional pork meat. The material consisted of three elements of pork meat: loin, ham, shoulder. The meat, intended for consumer market, was bought in the same, organically certified slaughterhouse, which enabled the elements to be cut out fresh from the same carcass. Eighteen meat samples (organic and conventional) were selected. The samples were vacuum-packed and transported, in refrigerated conditions, without any exposure of light, directly to the analytical laboratory. The analysis of the samples was carried out 24 h after the slaughter. Before the analysis, the whole-muscle samples were purified by removing external fat and membranes. The experiment was repeated three times. The research aimed at simultaneous verification of all the conditions connected to farming (organic/conventional), as well as their effect on the difference in protein degradation and formation of bioactive peptides with antioxidant activity from meat available for consumers. However, an interview conducted with the manager of the slaughterhouse made it possible to determine the following:

- The pigs were crossbreed (wbp × pbz);
- The first group was reared in conventional stables, while the other group was reared in special stables, arranged according to the Commission Regulation (EC) No. 889/2008 of 5 September 2008 [31], following the detailed rules of Council Regulation (EC) No. 834/2007 [32] on organic production and labeling of organic products with regard to organic production, labeling, and control;
- The conventional rearing system comprised an indoor area of 1 m^2^, whereas the organic rearing system had an indoor area with organic saw dust of 1 m^2^ and an outdoor area with free ranges of 1 m^2^. The conventional stable had a climate control system, and the organic stable had only gravitation ventilation.
- The organic feed consisted exclusively of raw materials from organic farms and was produced directly on the farm. The second group of animals was fed with commercial feeds available for conventional producers. The composition and nutritional value of the compound feed were in line with the pig nutrition standards of the National Research Council [33]. Both conventionally and organically reared animals were given their diets and water ad libitum.
- One day before slaughter, the hogs (weighing approximately 110 kg) were transported to the abattoir, where they were rested overnight with free access to water. The animals were conventionally slaughtered after an electrical stunning.

### 2.2. Peptide Extraction and LC–MS/MS Identification

The peptides were isolated from the meat samples according to the procedures provided by Mora et al. [13]. Briefly, muscles (15 g) were homogenized with 100 mL of 0.01 N HCl for 5 min. The homogenate was centrifuged (2200 rpm for 20 min at 4 °C). The supernatant was decanted and filtered through glass wool, and then deproteinized by adding three volumes of ethanol and centrifuged, retaining the previously defined conditions. The obtained supernatant was then dried in a vacuum evaporator (Rotavapor R-215, BüchiLabortechnik AG, Flawil, Switzerland). The dried extract was dissolved in 0.01 N HCl, filtered through a 0.45 μM nylon membrane filter (Millipore, Bedford, MA, USA) and stored at −60 °C prior to further use. The peptides were analyzed by liquid chromatography coupled with tandem electrospray mass spectrometry (LC–MS/MS). The samples were concentrated and desalted on an RP-C18 pre-column (Waters Corp., Milford, MA, USA), and further peptide separation was achieved on an RP-C18 nano-Ultra Performance column (Waters) using a 180 min linear acetonitrile gradient (0–35%) at a flow rate of 250 nL·min^−1^. The outlet of the column was directly connected to a mass spectrometer (Orbitrap Velos, Thermo Fisher Scientific Inc. Waltham, MA, USA). The raw data files were preprocessed using Mascot Distiller software (version 2.4.2.0, Matrix Science Inc., Boston, MA, USA). The obtained peptide masses and their fragmentation pattern were compared with the protein sequence database (UniProt KB, www.uniprot.org) using the Mascot search engine (Mascot Daemon v. 2.4.0, Mascot Server v.2.4.1, Matrix Science, London, UK). The “mammals” option was chosen as the taxonomy constraint parameter. The following search parameters were applied: enzyme specificity, none; peptide mass tolerance, 5 ppm; fragment mass tolerance, 0.01 Da. The protein mass was left unrestricted, and mass values were monoisotopic with a maximum of two missed cleavages allowed. Methylthiolation, oxidation, and carbamidomethylation were set as fixed and variable modifications. The sequences of peptides from unknown original proteins were not listed. The peptide identification was performed using the Mascot search engine (Matrix Science), with a probability-based algorithm. An expected value threshold of 0.05 was used for the analysis (all peptide identifications had less than 0.05% chance of being a random match).

### 2.3. The Identification of Bioactive Peptides—In Silico Analysis

The peptides identified in meat samples were investigated as a source of bioactive peptides in relation to the information about peptides previously identified in the literature, using the BIOPEP-UWM database (http://www.uwm.edu.pl/biochemia/index.php/pl/biopep; access: December 2019) [34].

### 2.4. Evaluation of Bioactive (Antioxidant) Peptides—In Vitro Analysis

#### 2.4.1. Ability to Scavenge 2,2′-Azino-bis-3-ethylbenzthiazoline-6-sulfonic Acid (ABTS) 

The ability of the extracts obtained to scavenge free radicals was tested using the method of Re et al. [35] with the free-radical ABTS. The degree of reduction of ABTS^•^ was determined spectrophotometrically at 734 nm. The scavenging ability was determined using the following formula:

ABTS radical-scavenging activity (%) = (1 − A_2_/A_1_) × 100,

where A_1_ is the absorbance of the control sample, and A_2_ is the absorbance of the sample.

#### 2.4.2. Ability to Chelate Fe(II) Ions

The study on the ability to chelate Fe(II) ions by compounds contained in sample extracts was conducted according to the method of Decker and Welch [36]. The absorbance of the colored complex was measured spectrophotometrically at 562 nm.

Fe(II)-chelating activity (%) = (1 − A_2_/A_1_) × 100,

where A_1_ is the absorbance of the control sample, and A_2_ is the absorbance of the sample.

#### 2.4.3. Fe(III) Reduction Power (FRAP)

The FRAP method according to Oyaizu [37] involves the reduction of the reagent (Fe(III)) in stoichiometric excess relative to antioxidants. A spectrophotometric method with measurements at 700 nm was used. A higher absorbance value indicates a higher ability to reduce the test substance.

### 2.5. Statistical Analysis

Statistical analysis was carried out using Statistica 13.1 (StatSoft, Cracov, Poland) and Microsoft Office Excel 2013 software. Based on a two-factor analysis of variance (meat element, rearing system), homogeneous groups were separated using the Tukey test. The differences were considered statistically significant at *p* < 0.05. All of the data were presented as mean ± standard error. In the case of multidimensional analyses, a hierarchical grouping of data based on identified peptides was performed, and the results were presented as a heat map and dendrogram, using the Ward method and Euclidean distance. Data were normalized prior to the analysis.

## 3. Results

### 3.1. Protein Degradation and Peptide Formation

In this study, organic vs. conventional pork and the culinary element of the pork carcass (ham, loin, or shoulder) were compared, taking into account protein stability and peptide formation. Potential differences in the abundance of endogenous peptides were evaluated with an LC–MS-based peptidomic approach. In total, 4178 peptide sequences were identified. The analyses were performed in triplicate, and only the sequences present in three independent replicates were selected for further analysis. Eventually, 646 peptides were chosen for the subsequent stages of the analysis.

**Figure 1:**
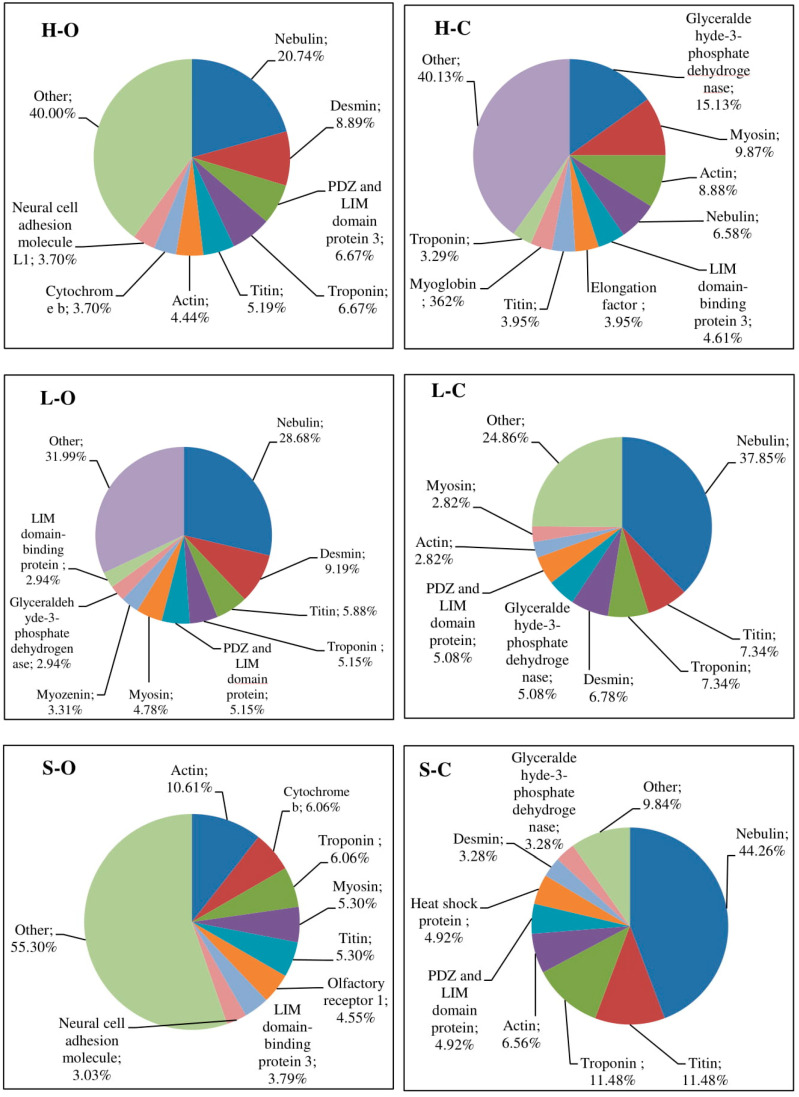
Distribution in percentages of identified peptides according to the origin of proteins in the analyzed muscle tissue (abbreviations: H-O: organic ham, L-O: organic loin, S-O: organic shoulder, H-C: conventional ham, L-C: conventional loin, S-C: conventional shoulder).

The distribution of identified peptides with regard to their protein origin is shown in Figure 1. A diverse number of protein fragments were identified, with no clear tendency depending on the type of muscle. Nevertheless, Picard et al. demonstrated a relationship between the protein profile and the muscle type [38]. The authors revealed relationships between proteins differing in contractile and metabolic properties (acting as biomarkers of tenderness and intramuscular fat content) compared in five bovine muscles [38]. According to Picard and Gagaou, a muscle-type effect depends on the biological function of proteins [39]. The authors emphasized that, in longissimus thoracis muscle, tenderness-related proteins mainly correspond to contractile, structural, and heat-shock proteins, while, in semitendinosus muscle, the tenderness-related proteins are mainly involved in metabolism. In the present study, the rearing system did not affect the protein profile distinctly. This observation is consistent with descriptions by Picard et al., who reported that the abundance of very few proteins from bovine meats was modified by rearing practices [40]. Moreover, factors associated with diet composition had weak effects on protein abundances determined by proteomics [39]. These data indicate that the impact of rearing practices on the proteome is muscle-type-dependent. As expected, several significant proteins were identified. Protein chains were degraded to produce a large number of different peptides, which confirmed proteolytic activity postmortem.

The highest diversity of proteins as peptide precursors was obtained in organically produced shoulder meat, where the highest percentage of the most different proteins (named “other” according to the designations in Figure 1) was classified. The protein generating the highest number of peptides was nebulin (in order, conventionally produced shoulder meat (44.26%) > conventional loin (37.85%) > organic loin (28.68%) > organic ham (20.74%) > conventional ham (6.58%)). Other myofibrillar proteins, especially structural proteins such as desmin, titin, or troponin were also the main sources of peptides in the analyzed meat samples (Figure 1). According to the literature, myosin, nebulin, actin, tropomyosin, tropomodulin, and troponin (assigned a role in stabilizing and determining highly ordered muscle structure), and nebulin, tropomyosin, and tropomodulin (“protein rulers” to precisely regulate the connection of myosin and actin fibers) are very sensitive to proteolysis [41].

Previous studies of postmortem protein degradation in pig muscle, using peptide profiling and amino-acid sequence analysis of protein fragments, showed that fragments of myofibrillary protein primarily undergo postmortem degradation, before severe deterioration catalyzed by cathepsins takes place. Taylor et al. reported that nebulin, titin, vinculin, and desmin are significantly degraded within three days of death in the semimembranosus muscle, which is consistent with the present report [42]. Furthermore, Huff Lonergan reported the postmortem µ-calpain-induced degradation of titin, nebulin, filamin, desmin, and troponin-T from bovine longissimus thoracis [43,44]. Sarcoplasmic proteins (among others, glyceraldehyde-3-phosphate dehydrogenase, myosin, and PDZ (PSD95, Dig, ZO-1) and LIM (Lin11, Isl-, Mec-3) domain protein-3) gave smaller amounts of peptides relative to myofibrillary proteins (Figure 1). Previous studies consistently showed that myofibrillar proteins were more easily hydrolyzed than sarcoplasmic proteins during postmortem meat tenderization. Nevertheless, sarcoplasmic protein fragments similar to those from the present study were also reported as occurring in tenderization processes [45,46].

Various methods of analyzing meat tissue were presented in the literature, regarding the presence of tissue-specific proteins, as well as their proteolysis or denaturation due to storage and processing. As an example, Sarah et al. discovered porcine-specific peptides as potential markers for meat species using LC–QTOF-MS [47]. In addition, Kim et al. [48] proposed a proteomic method for the authentication of meat species such as raw beef, pork, and poultry (chicken and duck), using a protein-based approach, including one-dimensional (1D) gel electrophoretic separation and LC–MS/MS analysis. The authors showed that troponin I, enolase 3, 1-lactate dehydrogenase, and triose phosphate isomerase may be useful markers for distinguishing mammalian meat from poultry [48]. They also emphasized that species-specific peptides determined by LC–MS/MS allow each species to be identified independently from the same protein. Furthermore, Mi et al. [49] defined the differences between Tibetan and Duroc (Landrace × Yorkshire) pork, using a label-free quantitative proteomics approach. Therefore, in the present study, an attempt was made to assess whether specific peptides show a difference between different elements of pork carcasses, but from the same species. The impact of conventional or organic pork origin was also considered. The general aim of this approach consisted of profiling the changes in peptide composition. The final peptide composition was identified, and, among the selected peptides, only five identical peptides from muscle tissue proteins occurred in each variant analyzed (Table 1), suggesting large differences in the peptide profiles. 

**Figure 2:**
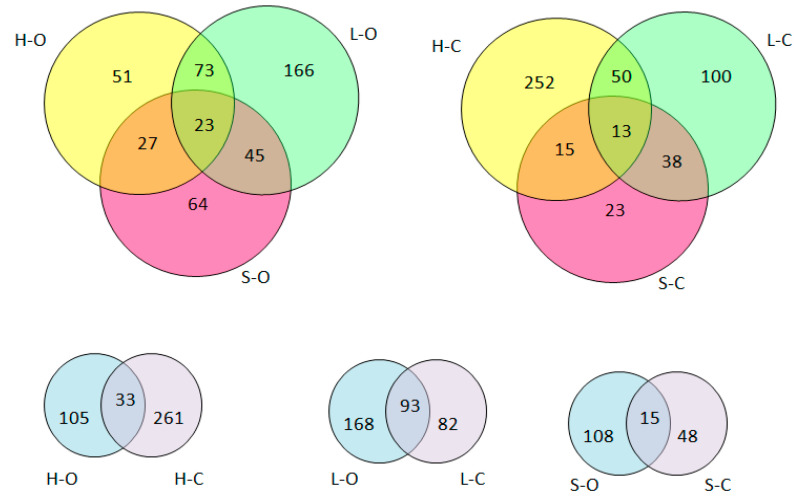
Venn diagram showing number of peptides obtained in the pork meat tissue (abbreviations: H-O: organic ham, L-O: organic loin, S-O: organic shoulder, H-C: conventional ham, L-C: conventional loin, S-C: conventional shoulder).

A qualitative comparison of the unique and common peptides identified in the three muscle types is shown in the Venn diagrams (Figure 2). The analyzed samples had a different peptide profile, depending on the meat origin (conventional vs. organic). The obtained LC–MS/MS spectra made it possible to identify the peptides characteristic for conventional (Table 2) or organic (Table 3) meat.

In addition, the samples collected from different elements of pork carcasses had a different peptide profile (Tables S1–S3, Supplementary Materials). The highest diversity of peptides was obtained for ham samples, characterized by the highest number of individual sequences (105 peptides and 261 peptides for organic ham and conventional ham, respectively) with a low number of common sequences (i.e., 33 peptides). The most similar analysis results were obtained for loins, for which 93 common peptide sequences were determined. Considering the pork origin (organic vs. conventional), there was no clear trend in the number of peptides. A higher variety of peptides, characteristic for the element of pork carcasses, was noted for the organic meat samples (organic loin, 166 peptides; organic shoulder, 64 peptides), while the smallest number of specific sequences (typical for these samples only) was obtained for organic ham (i.e., 51 peptides). 

**Figure 3:**
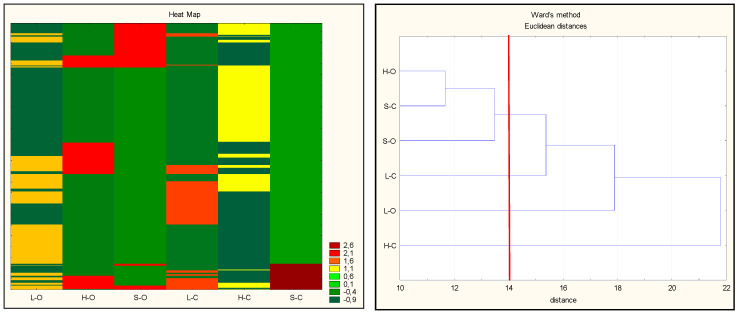
Heat map of abundance levels (**left**) and dendrogram (**right**) obtained as a result of hierarchical clustering based on proteomic data. The changes in abundance of statistically significant (*p* < 0.05) spots among meat tissue models were analyzed. Fold change: negative values (decreasing abundance), 0 (no differences), positive values (increasing abundance); abbreviations: H-O: organic ham, L-O: organic loin, S-O: organic shoulder, H-C: conventional ham, L-C: conventional loin, S-C: conventional shoulder.

The resulting dataset was also used to assess the similarity between the samples, using multidimensional hierarchical clustering of objects. For data visualization, a heat map with a color scale was introduced to encode existing values from the smallest to the largest (a higher color intensity at each scale represents a higher change in the number of each place). The highest intensity of green color (and, hence, the smallest abundance of given peptide sequences) was characterized for conventional ham and organic loin, which corresponds to the results presented in Figure 2. These batches contained the largest number of individual, characteristic peptide sequences. The highest values marked on the heat map in red were obtained for conventional shoulder, followed by organic ham and organic shoulder, indicating the smallest diversity of peptide sequences among all analyzed variants. These assays simultaneously formed a common cluster on the dendrogram shown in Figure 3.

Differences in the peptide content between particular muscle types may probably result from their chemical composition. Oxidative muscles generally contain more lipids than glycolytic muscles. As reported by Bonnet et al., the abundance of myosine-1- or triosephosphate isomerase was appropriately distinguished between the lean or fat muscle groups observed, when using proteomics [50]. Bazile et al. identified proteins with abundance differing depending on carcass and muscular dispositions in longissimus thoracis from cows [51]. Seven proteins involved in glycolysis or gluconeogenesis were the least abundant, while 14 proteins related to oxidative metabolism, slow-type muscle, or retinoic acid metabolism were the most abundant in the high-adiposity group.

### 3.2. Antioxidant Properties of Peptides—In Silico Analysis

**Figure 4:**
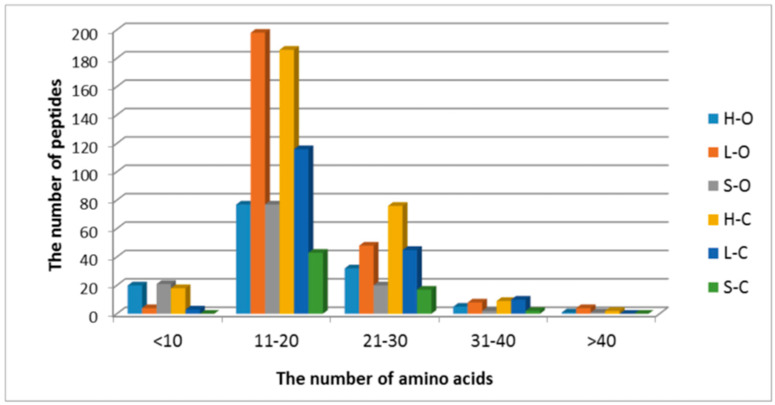
The distributions of peptides based on their molecular weight (abbreviations: H-O: organic ham, L-O: organic loin, S-O: organic shoulder, H-C: conventional ham, L-C: conventional loin, S-C: conventional shoulder).

**Figure 5:**
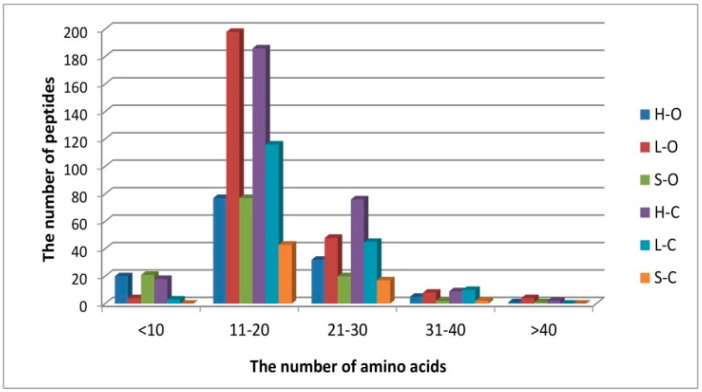
The distributions of peptides based on their number of amino-acid residues (abbreviations: H-O: organic ham, L-O: organic loin, S-O: organic shoulder, H-C: conventional ham, L-C: conventional loin, S-C: conventional shoulder).

In this study, peptides were identified with high precision and a mass tolerance lower than 5 ppm. The length of identified peptides ranged from 7–50 amino acids (50 amino acids: only one peptide from creatine kinase at chain position 331–381, M-type; Uniprot ID Q5XLD3, data not shown). The number and type of peptide sequences were compared depending on the type of carcass element and rearing system, and the results are presented graphically in Figure 4 and Figure 5.

Short-chain peptides had higher antioxidant activity than their proteins and polypeptides of origin, as also suggested by Zhu et al. [52]. A common feature of antioxidant peptides is 4–16 amino acids and a molecular weight of about 400–2000 Da [19]. In the present study, sequences predominantly contained 11–20 amino acids (Figure 4), with a molecular weight ranging from 1500 to 2000 Da (Figure 5). 

The obtained peptide sequences were evaluated with regard to a potential biological activity using an in silico approach. In particular, the antioxidant potential properties of peptides were considered. To reduce the search area, peptides common for the muscle types tested (Supplementary Materials) and the meat origin (conventional vs. organic; Table 2 and Table 3, respectively) were selected for further analysis. 

The main purpose of the in silico approach was to determine the antioxidant capacity of meat peptides. However, among the analyzed sequences, low levels of antioxidant peptides were recognized. Only a few peptide sequences, such as dipeptides (AY, AH, EL, HH, HL, KD, IR, KP, LK, LY, LH, MM, SE, TW, WY, VY) and tripeptides (LHV, IKK, VKL, VKV, PEL, PHQ, SDF, FVP, GAA, GAH), as well as one four-amino acid sequence (YVGD) were found. Their location and source of origin are presented in Table 3 and Table 4, Tables S1–S3. Nevertheless, in this study, peptides acting as a potential antioxidant were obtained primarily from myofibrillary proteins (nebulin and rarely titin, regardless of the rearing system applied). Kęska and Stadnik [14] indicated higher values of the in vitro antioxidant activity of myofibrillary proteins compared to water-soluble (sarcoplasmic) proteins in the ABTS test in dry-cured pork loin. These results are in line with the previous observations that the total number of bioactive peptides predicted to be released after in silico pepsin or pancreatin hydrolysis of selected porcine myofibrillar proteins ranged from six peptides for troponin C, skeletal muscle troponin C (TNNC2) to 112 for myosin-2 Myosin-2 (MYH2), preceded by nebulin (NEB) with 109 peptides per protein molecule. Of these, 1, 13, and 11 two- or three amino-acid peptides with antioxidant properties were observed (for TNNC2, MYH2, and NEB, respectively) [20].

Some food-derived peptides were reported to be multifunctional, because they can provide two or more health-promoting effects. As shown in Table 2 and Table 3, the analyzed peptides may have more than one bioactivity, mainly acting as dipeptidyl peptidase-IV (DPP-IV) and angiotensin-I converting enzyme (ACE-I) inhibitors. Both groups of biologically active peptides can act against the effects of non-communicable diseases. The DPP-IV inhibitors belonging to one of these groups are involved in the regulation of blood glucose levels and are, therefore, strong antidiabetic agents. A similar bioactive action attributed also to another group of peptides termed “glucose uptake-stimulating peptides” was detected in this study. In turn, the ACE-I inhibitor, by inhibiting the conversion of angiotensin, reduces the negative effects of hypertension. All peptides analyzed in this study can act as DPP-IV inhibitors as well as ACE-I inhibitors (except for one peptide sequence (V.IIIIIIIIII.I), as shown in Table 3). As an example, based on the in silico analysis, dipeptide AY has antioxidant, cardioprotective, and antidiabetic effects. In addition, the potential of proteins as precursors of dipeptidyl peptidase-III (DPP III) inhibitors was also noted. The peptidase with DPP-III-inhibiting activity has a high affinity for cleavage of opioid peptides such as endomorphins and encephalin. These opioid peptides regulate a variety of physiological functions, such as signal transduction, gastrointestinal motility, immune and hormonal functions, and pain modulation. 

According to the report by Khaket et al. [53], the β-subunit of chicken hemoglobin and annexin A5 showed a high inhibitory potential for DPP-III in in silico studies. However, as noted by Galleo et al. [16], only a few studies identified peptides that inhibit DPP-III from meat proteins. Therefore, the information gathered in this study regarding pork as a potential source of DPP-III is of particular interest. The presence of these biopeptides was identified in nebulin (YK, DA, KA, IH, PE, LR MR, RV), as well as in astitin (RV) and desmin (GE) (Table 2 and Table 3). Furthermore, several peptides with biologically active properties, such as renin inhibition, calmodulin-dependent phosphodiesterase (CaMPDE) inhibition, dipeptidyl carboxypeptidase inhibition, antiamnestic and antithrombotic activity, regulation of the stomach mucosal membrane activity, bacterial permease ligand ability, and activation of ubiquitin-mediated proteolysis, were reported in unique biopeptides (Table 2 and Table 3).

### 3.3. Antioxidant Properties of Peptides—In Vitro Analysis

The effectiveness of antioxidant compounds depends on various mechanisms. Consequently, a single test cannot cover all of the different modes of action of different food systems, especially in complex tissues such as meat. Therefore, three different tests were used to assess the differences between conventional and organic meats, as well as between selected pork elements. The variance analysis indicated that all of the effects of the rearing system or muscle type on the total antioxidant activity were significant (*p* < 0.05). The obtained values of radical-scavenging activity measured by the ABTS test were similar, ranging from 41.89% for organic loin to 33.92% for organic shoulder. The rearing method did not significantly affect the radical-scavenging activity of peptides in the ABTS test. This observation is also consistent with other descriptions in the literature, where meat extracts were also analyzed [27,28]. However, the meat origin (organic vs. conventional) and the carcass element significantly affected the iron-ion-chelating capacity (Fe(II)) and the reducing power. Descalzo et al. [54] reported that meat samples from animals reared on pasture had a higher content of antioxidant vitamins (α-tocopherol, β-carotene, and ascorbic acid) than the meat obtained from grain-fed animals. This observation became the basis for estimating the antioxidant potential of meat samples from various feeding systems. The authors further showed that the pasture rearing system had a greater reduction potential than in the case of grain-fed animal samples according to the FRAP assessment; however, there were no differences between these groups in the ABTS test. Importantly, the results indicated the non-enzymatic antioxidants as a cause of differences in antioxidant properties in the samples of meat from animals reared on pasture or fed on grains [28].

As presented in Table 4, the antioxidant properties were higher in the conventional meat samples, while the largest differences were noted in ham samples, i.e., 4.46% for the iron chelate efficiency (*p* < 0.05) and ΔA_700_ = 0.175 for reducing power activity (*p* < 0.05). Based on the results of the FRAP tests, the smallest differences between organic and conventional pork were noted for shoulder batches.

**Figure 6:**
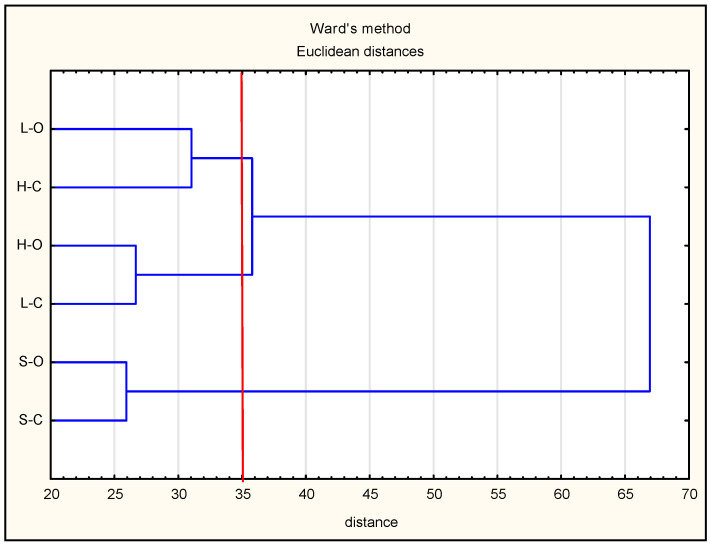
Dendrogram resulting from Ward’s method of hierarchical cluster analysis of antioxidant activity.

To better understand how the carcass elements (ham, loin, or shoulder) collected from animals from a conventional or organic farming system were associated with the presence of antioxidant peptides, the changes in their antioxidant properties were quantified using various tests. By means of hierarchical clustering, the peptides were grouped according to their antioxidant trends. The hierarchical cluster analysis (HCA) was used in this study to calculate the multidimensional Euclidian distances between the observations (antioxidant activity). Using a stepwise algorithm (Ward’s linkage criterion), observations behaving similarly across the initial variables were linked, and the results were graphically shown in a clustering tree (Figure 6).

The groups of observations behaving similarly were gathered in clusters. The derived dendrogram made it possible to distinguish three groups: cluster 1, organic loins and conventional ham; cluster 2, organic ham and conventional loins; cluster 3, organic and conventional shoulders (the most separated group). As observed in this study, conventional ham and organic loin were characterized by the highest variety of peptides. This confirms the hypothesis that the quantity and quality of peptides determine their contribution to act as additional antioxidant compounds against oxidation. On the basis of the results obtained, it can be stated that the antioxidant activity of peptides from pig muscles corresponds to their specific (individual) sequences.

As the role of food-derived antioxidants is significant, research on them is still widely carried out. Despite the fact that studies on living organisms are important in verifying the biological properties of peptides, there are little data on the direct antioxidant potential of food peptides on cell biology at the living organism level. So far, there have only been a few studies on food-origin antioxidant peptides in animal models. The administration of egg-white hydrolysate to spontaneously hypertensive rats for 17 weeks was shown to improve plasma antioxidant properties [55]. In turn, Ebaid et al. [56] showed that administering 100 mg/kg body weight of whey protein to streptozotocin-induced diabetic rats reduced several indicators of oxidative stress, such as malondialdehyde, nitric oxide, and ROS levels, while it also reduced proinflammatory cytokines and increased the level of glutathione [56]. Cellular assays are more commonly used as indirect methods to assess the protective effect of antioxidants against oxidative stressors and to elucidate the mechanism of action of peptides in cells. Thus, Katayama, Xu, Fan, and Mine, using human intestinal epithelial cells (Caco-2) as an intestinal epithelia model, reported that oligophosphopeptides derived from hen egg yolk exert an antioxidant effect, causing the upregulation of glutathione-induced biosynthesis accompanied by increased glutathione reductase activity [57]. This observation was also accompanied by inhibition of the production of proinflammatory cytokines, contributing to antioxidative protection against hydrogen peroxide-induced damage in Caco-2 cells. Moreover, the antioxidant peptide from fish skin gelatin hydrolysate increased the expression of cellular antioxidant enzymes (catalase, superoxide dismutase, and glutathione peroxidase) in human hepatoma (Hep3B) cells [58]. Numerous reports showed that food-derived peptides can retain antioxidant activity in simulating gastrointestinal digestion and then retain their properties in further studies of the cellular pathway (as a simulated uptake step) in the Caco-2 cell monolayer [59,60,61]. Research results confirmed that peptides from food can cross the gastrointestinal barrier and exert antioxidant effects. Thus, upon oral ingestion of peptide-rich foods, such as meat, different in vivo antioxidant efficacy can be produced.

## 4. Conclusions

The obtained results indicate some influence of the meat origin (organic vs. conventional) on the health characteristics of pork. Moreover, an in-depth mapping of the peptidome of pig carcass elements obtained from these two farming methods provides (at the molecular level of the protein) important information on how the peptide profile is being shaped. Based on the LC–MS/MS analysis of the different carcass elements, myofibrillary proteins (such as nebulin, titin, or desmin) were identified as the main sources of peptides. However, the meat origin (organic vs. conventional), as well as the element of the pork carcass (ham, loin, or shoulder), did not have a significant effect on the proteome. On the other hand, the pork origin and the carcass element significantly affected the iron-ion-chelating capacity (Fe(II)) and the reducing power of peptides. In particular, pork ham from the conventional rearing system, which had the best antioxidant properties due to the presence of peptides, can be recommended for daily consumption to people who care about their health. Analyses also showed that this meat element may be a source of peptides that support the treatment of noncommunicable diseases such as hypertension and diabetes, but additional research is needed to further confirm this aspect.

## Figures and Tables

**Table 1:** Peptide sequences identified in all analyzed samples.

Lp	Sequence ^1^	Protein	Position ^2^
1	R.VREPVISAVEQTAQR.T	Titin	[438–452]
2	T.IEPDAVHIKAAKDAYK.V	Nebulin	[5535–5550]
3	E.EAPPPPAEVHEVHEEVH.E	Troponin T, fast skeletal muscle	[21–38]
4	W.ITKQEYDEAGPSIVHRK.C	Actin, alpha-skeletal muscle	[358–375]
5	M.WITKQEYDEAGPSIVHRK.C		[357–375]

^1^ The period indicates cutting points; ^2^ position in parental protein.

**Table 2:** List of peptide sequences common for conventional meat. ACE: angiotensin-I converting enzyme; DPP: dipeptidyl peptidase.

	Sequence ^1^	Protein	Position ^2^	ACE Inhibitor	DPP-IV Inhibitor	Stimulating ^3^	Antioxidant
1	R.VREPVISAVEQTAQR.T	Titin	[438–452]	VR, VE, AV	EP, TA, VR, AV, PV, QT, VE, VI	-	-
2	S.VVDTPEIIHAQQVKN.L ^4^	Nebulin	[6063–6077]	VK, EI, TP	VV, HA, TP, EI, IH, II, QQ, QV, VD, VK,	II	-
3	T.IEPDAVHIKAAKDAYK.V ^5^	Nebulin	[5525–5540]	AY, AA, DA(2) ^6^, YK, KA, IE, AV, IEP	KA, EP, AA, AV, AY, HI, VH, YK	-	AY, KD
4	Q.IRKETEKAFVPKVVIS.A ^7^	Titin	[512–527]	IR, AF, VP, KA, TE, EK, KE, VPK, FVP	KA, VP, VV, EK, AF, ET, IR, KE, KV, PK, RK, TE, VI	-	IR
5	E.EAPPPPAEVHEVHEEVH.E ^8^	Troponin T, fast skeletal muscle	[22–38]	AP, EA, EV(3), EAP, PPP(2)	PPPP, PP(3), AP, PA, AE, EV(3), HE(2), VH(2)	EE	-
6	M.ARVREPVISAVEQTAQR.T ^9^	Titin	[436–452]	VR, AR, VE, AV, RVR	EP, TA, VR, AV, PV, QT, VE, VI	-	-
7	T.IETRDGEVVSEATQQQH.E ^10^	Desmin	[452–468]	GE, EA, DG, IE, EV, TQ,	VV, AT, ET, EV, GE, QH, QQ(2), TQ, TR, VS,	SE	-
8	W.ITKQEYDEAGPSIVHRK.C ^11^	Actin, alpha-skeletal muscle	[359–375]	GP, AG, EA, EY, AGP	GP, AG, EY, HR, PS, QE, RK, SI, TK, VH, YD	IV	-
9	N.FTSVVDTPEIIHAQQVKN.L ^12^	Nebulin	[6060–6077]	VK, EI, TP	VV, HA, TP, EI, HI, II, QQ, QV, SV, TS, VD, VK	II	-
10	N.YKADLKDLSKKGYDLRTD.A ^13^	Nebulin	[1052–1069]	GY, KG, YK, KA, LR	KA, AD, GY, KG, KK, SK, TD, YD, YK	-	KD, LK
11	M.WITKQEYDEAGPSIVHRK.C ^14^	Actin, alpha-skeletal muscle	[358–375]	GP, AG, EA, EY, AGP	GP, WI, AG, EY, HR, PS, QE, RK, SI, TK, VH, YD	IV	-
12	A.AVDMARVREPVISAVEQTAQR.T ^15^	Titin	[432–452]	VR, AR, VE, AV(2), DM, RVR	MA, EP, TA, VR, AV(2), PV, QT, VD, VE, VI	-	-
13	L.YKEDVSPGTAIGKTPEMMRVKQTQDH.I ^16^	Nebulin	[6154–6179]	VK, IG, GK, GT, PG, YK, TQ, KE, AI, TP, MM, VSP	TP, SP, TA, KE, KT, MM, MR, PG, QD, QT, TQ, VK, VS, YK	-	MM

^1^ Periods indicate cutting points; ^2^ position in parental protein; ^3^ stimulating vasoactive substance release or glucose uptake-stimulating peptide; ^4^ other activity: DPP-III inhibitor, IH, PE; ^5^ other activity: DPP-III inhibitor, YK, DA (2) 6, KA; ^6^ the number in parentheses indicates the number of identified peptides if more than one; ^7^ other activity: CaMPDE (calmodulin-dependent phosphodiesterase) inhibitor and renin inhibitor, IR; DPP-III inhibitor, KA; ^8^ other activity: dipeptidyl carboxypeptidase inhibitor, PPPA; ^9^ other activity: DPP-III inhibitor, RV; ^10^ other activity: DPP-III inhibitor, GE; ^11^ other activity: antiamnestic (prolyl endopeptidase inhibitor) and antithrombotic, GP; regulating stomach mucosal membrane activity, GP; ^12^ other activity: DPP-III inhibitor, PE; rennin inhibitor, FT; ^13^ other activity: bacterial permease ligand, KK; DPP-III inhibitor, LR, YK, KA; rennin inhibitor, LR; ^14^ other activity: antiamnestic (prolyl endopeptidase inhibitor), antithrombotic, and regulating stomach mucosal membrane activity, GP; ^15^ other activity: DPP-III inhibitor, RV; ^16^ other activity: antiamnestic (prolyl endopeptidase inhibitor), antithrombotic, and regulating stomach mucosal membrane activity, PG; DPP-III inhibitor, MR, YK, RV, PE.

**Table 3:** List of peptide sequences common for organic meat.

	Sequence ^1^	Protein	Position ^2^	ACE Inhibitor	DPP-IV Inhibitor	Stimulating ^4^	Antioxidant
1	V.IIIIIIIIII.I	Killer cell immunoglobulin-like receptor	[341–349]	-	II (9) ^3^	II (9)	-
2	A.IILLLLILLI.L	Neural cell adhesion molecule	[1132–1142]	IL(2)	LL(4), II, IL(2), LI(2)	LLL(2), IL(2), LI(2), II, LL(4)	-
3	I.ILLLLILLIL.C	Neural cell adhesion molecule	[1133–1143]	IL(3)	LL(4), IL(3), LI(2)	LLL(2), IL(3), LI(2), LL(4)	-
4	S.ILILLIILLL.H	Cytochrome b	[297–307]	IL(3)	LL(3), II, IL(3), LI(2)	LLL(1), IL(3), LI(2), II, LL(3)	-
5	V.ILLLLLLLLL.F	Leukocyte immunoglobulin-like receptor subfamily B	[469–479]	IL(2)	LL(5), IL(2), LI(2)	LLL(4), IL(2), LI(2), LL(5)	-
6	F.LILILLLLLL.V	Cadherin-1	[731–741]	IL(2)	LL(5), IL(2), LI(2)	LLL(4), IL(2), LI(2), LL(5)	-
7	G.LLILILLLLL.L	Cytochrome b	[232–241]	IL(2)	LL(5), IL(2), LI(2)	LLL(3), IL(2), LI(2), LL(5)	-
8	A.LLLILILLLL.V	Cytochrome b	[233–242]	IL(2)	LL(5), IL(2), LI(2)	LLL(3), IL(2), LI(2), LL(5)	-
9	H.LLLLLLIIIL.T	Myeloma-overexpressed gene protein	[302–311]	IL(2)	LL(5), II(2), IL, LI	LLL(4), IL, LI, II(2), LL(5)	-
10	C.LLLLLLLLIL.R	Ephrin	[186–195]	IL	LLL(6), IL, LI, LL(7)	LL(7), IL, LI	-
11	P.PPPAEVHEVHEEVH.E ^5^	Troponin T, fast skeletal muscle	[25–38]	EV(3), PP(2), PPP	PP(2), PA, AE, EV(3), HE(2), VH(3)	EE	-
12	R.VREPVISAVEQTAQR.T	Titin	[437–452]	VR, VE, AV	EP, TA, VR, AV, PV, QT, VE, VI	-	-
13	S.VNVDYSKLKKEGPDF ^6^	Cytochrome c oxidase subunit NDUFA4	[68–82]	GP, EG, KL, KE, DY, DF	GP, EG, KE, KK, NV, SK, VD, VN, YS	-	LK
14	T.IEPDAVHIKAAKDAYK.V ^7^	Nebulin	[5525–5540]	AY, AA, DA(2), YK, KA, IE, IEP, AV	KA, KD, AA, AV, AY, HI, VH, YK,	-	AY, KD
15	E.APPPPAEVHEVHEEVH.E ^8^	Troponin T, fast skeletal muscle	[23–38]	AP, EV(3), PP(3)	PPPP, PPP(3), AP, PA, AE, EV(3), HE(2), VH(3)	EE	-
16	I.TKQEYDEAGPSIVHRK.C ^9^	Actin, alpha-skeletal muscle	[360–375]	GP, AG, EA, EY, AGP	GP, AG, EY, HR, PS, QE, RK, SI, TK, VH, YD	IV	-
17	L.KVSILAAIDEASKKLNAQ ^10^	Apolipoprotein A-I	[248–265]	LAA, LA, AA, EA, KL, LN, AI, IL	LA, AA, AS, IL, KK, KV, LN, NA, SI, SK, VS	IL	-
18	E.EAPPPPAEVHEVHEEVH.E ^11^	Troponin T, fast skeletal muscle	[22–38]	AP, EA, EV(3), PP(3), EAP, PPP(2)	PPPP, PP(3), AP, PA, AE, EV, HE, VH	EE	-
19	E.KAKDIEHAKKVSQQVSK.V ^12^	Nebulin	[153–169]	AKK, KA, IE	KA, HA, EH, KK, KV, QQ, QV, SK, VS(2), KA	-	KD
20	W.ITKQEYDEAGPSIVHRK.C ^13^	Actin, alpha-skeletal muscle	[359–375]	GP, AG, EA, EY, AGP	GP, AG, EY, HR, PS, QE, RK, SI, TK, VH, YD	IV	-
21	ISKQEYDESGPSIVHRK ^14^	POTE ankyrin domain family member F		GP, SG, EY, SGP	GP, ES, EY, HR, PS, QE, RK, SI, SK, VH, YD	IV	-
22	M.WITKQEYDEAGPSIVHRK.C ^15^	Actin, alpha-skeletal muscle	[358–375]	GP, AG, EA, EY, AGP	GP, WI, AG, EY, HR, PS, QE, RK, SI, TK, VH, YD	IV	-
23	L.KPRPPPPPPAPPKEDVKEKIFQ.L ^16^	Titin	[11804–11836]	PR, VK, RP, AP, IF, PAP, PPK, KP, PP(6), EK, KE(2), PAPPK, RPP, FQ, PPP(4)	PPPP(3), PP(6), AP, PA, RP, KP, EK, FQ, KE(2), KI, PK, VK, PR,	-	KP

^1^ Periods indicate cutting points; ^2^ position in parental protein; ^3^ the number in parentheses indicates the number of identified peptides if more than one; ^4^ stimulating vasoactive substance release or glucose uptake-stimulating peptide; ^5^ other activity: dipeptidyl carboxypeptidase inhibitor, PPPA; ^6^ other activity: antiamnestic (prolyl endopeptidase inhibitor) and antithrombotic, GP; regulating, DY, GP; bacterial permease ligand, KK; ^7^ other activity: DPP-III inhibitor, YK, DA (2), KA; ^8^ other activity: dipeptidyl carboxypeptidase inhibitor, PPPA; ^9^ other activity: antiamnestic (prolyl endopeptidase inhibitor), antithrombotic, and regulating, GP; ^10^ other activity: bacterial permease ligand, KK; activating ubiquitin-mediated proteolysis, LA; DPP-III inhibitor, LA; ^11^ other activity: dipeptidyl carboxypeptidase inhibitor, PPPA; ^12^ other activity: bacterial permease ligand, KK; ^13^ other activity: antiamnestic (prolyl endopeptidase inhibitor), antithrombotic, and regulating stomach mucosal membrane activity, GP; ^14^ other activity: antiamnestic (prolyl endopeptidase inhibitor), antithrombotic, and regulating stomach mucosal membrane activity, GP; ^15^ other activity: antiamnestic (prolyl endopeptidase inhibitor), antithrombotic, and regulating stomach mucosal membrane activity, GP; ^16^ other activity: antithrombotic, PPK; dipeptidyl carboxypeptidase inhibitor, PPPA, PPAP.

**Table 4:** The antioxidant properties of peptides based on in vitro analysis. ABTS: 2,2′-azino-bis-3-ethylbenzthiazoline-6-sulfonic acid; FRAP: Fe(III) reduction power.

Antioxidant Properties		Ham	Loin	Shoulder	Rearing System (A)	Meat Element (B)	A × B
ABTS assay [%]	O	39.80 ± 3.11 ^Aa^	41.89 ± 3.48 ^Aa^	33.92 ± 3.97 ^Ab^	NS	***	***
	C	41.51 ± 3.21 ^Ba^	38.06 ± 2.69 ^Bb^	34.19 ± 1.71 ^Ac^			
Fe(II) assay [%]	O	12.91 ± 2.01 ^Aa^	13.49 ± 1.60 ^Aa^	13.24 ± 2.50 ^Aa^	***	*	**
	C	17.37 ± 1.41 ^Ba^	14.77 ± 1.73 ^Ab^	14.72 ± 1.37 ^Ab^			
FRAP assay	O	0.567 ± 0.024 ^Aa^	0.653 ± 0.028 ^Ab^	0.606 ± 0.011 ^Ac^	***	***	**
	C	0.742 ± 0.058 ^Ba^	0.767 ± 0.036 ^Ba^	0.767 ± 0.039 ^Ba^			

Abbreviations: O: organic rearing, C: conventional rearing. The results are presented as mean ± SD (standard deviation); ^a–c^ means in the same row with different letters differ significantly (*p* < 0.05); ^A, B^ means in the same column with different letters differ significantly (*p* < 0.05); NS, not significant; * *p* < 0.05, ** *p* < 0.01, *** *p* < 0.001.

## Supplementary Materials

The following are available online at https://www.mdpi.com/2076-3921/9/9/835/s1, Table S1. The list of peptide sequences common for conventional and organic ham; Table S2. The list of peptide sequences common for conventional and organic loins; Table S3. The list of peptide sequences common for conventional and organic shoulders.
