# Supplementary material for: Peptides from Different Carcass Elements of Organic and Conventional Pork—Potential Source of Antioxidant Activity
Source: Antioxidants (Basel). 2020 Sep 7;9(9):835. doi: 10.3390/antiox9090835 (PMC7554766; doi:10.3390/antiox9090835)
Supplement: Supplementary file 1 [file antioxidants-09-00835-s001.pdf]

Table 1. List of peptide sequences common for conventional and organic ham

	Sequence[1]	Protein	Position[2]	ACE inhibitor	DPP-IV inhibitor	Stimulating[3]	Antioxidant
**1**	G.LPEALIPVQLL.W[4]	Sarcoplasmic/endoplasmic reticulum calcium ATPase	[783-793]	IP, EP	LP,LL, IP, AL, LI, PV, QL, VQ	LI, LL	-
**2**	A.GAGIALNDHFVK.L[5]	Glyceraldehyde-3-phosphate dehydrogenase	[296-307]	VK, IA, GI, GA, AG, LN	GA, IA, AL, AG, GI, HF,LN, ND, VK	-	-
**3**	L.PPVVPVSKPGALR.R[6]	Telethonin	[141-153]	VP, GA, PG, KP, PP, LR	PP, VP, VV, KP, GA, AL, PG, PV, SK, VS	-	KP
**4**	S.LPVIGLDEILKF.V[7]	Sarcoplasmic/endoplasmic reticulum calcium ATPase	[975-986]	IG, GL, KF, EI, IL	LP, GL, EI, IL, KF, PV, VI	IL	LK
**5**	S.LPVIGLDELLKF.I[8]	Sarcoplasmic/endoplasmic reticulum calcium ATPase	[975-986]	IG, GL, KF	LP, LL, GL, KF, PV, VI	LL	EL, LK
**6**	E.LPDGQVITIGNER.F[9]	Actin, alpha skeletal muscle	[244-256]	IG, GQ, DG	LP, NE, QV, TI, VI	-	-
**7**	F.DAGAGIALNDHFVK.L	Glyceraldehyde-3-phosphate dehydrogenase	[294-307]	VK, IA, GA, GI, AG, DA, LN	GA, IA, AL, AG, GI, HF, LN, ND, VK, DA, HF	-	-
**8**	N.VDYSKLKKEGPDF,-[10]	Cytochrome c oxidase subunit NDUFA4	[70-82]	GP, EG, KL, KE, DY, DF	GP, EG, KE, KK, SK, VD, YS	-	LK
**9**	F.VAEPKESFVKGTVQS.R[11]	Myosin-1	[40-54]	VK, KG,GT, SF, KE	VA, EP, AE, ES, KE, KG, PK, QS, SF, TV, VK, VQ	-	-

**10**	R.VREPVISAVEQTAQR.T	Titin	[438-452]	VR, VE, AV	EP, TA, VR, AV, PV, QT, VE, VI	-	-
**11**	S.VVDTPEIIHAQQVKN.L[12]	Nebulin	[6063-6077]	VK, EI, TP	VV, HA, TP, EI, IH, II, QQ, QV, VD, VK	II	-
**12**	S.VNVDYSKLKKEGPDF.-[13]	Cytochrome c oxidase subunit NDUFA4	[68-82]	GP, EG, KL, KE, DY, DF	GP, EG, KE, KK, NV, SK, VD, VN, YS	-	LK
**13**	T.IEPDAVHIKAAKDAYK.V[15]	Nebulin	[5525-5540]	AY, AA, DA(2)[14], YK, KA, IE, IEP, AV	KA, EP, AA, AV, AY, HI, VH, YK	-	AY, KD
**14**	Q.IRKETEKAFVPKVVIS.A[16]	Titin	[512-527]	IR, VPK, AF, VP, KA, TE, EK, KE, FVP	KA, VP, VV, EK, AF, ET, IR, KE, KV, PK, RK, TE, VI	-	IR
**15**	I.TKQEYDEAGPSIVHRK.C[17]	Actin, alpha skeletal muscle	[360-375]	GP, AG, EA, EY, AGP	GP, AG, EY, HR, PS, QE, RK, SI, TK, VH, YD	IV	-
**16**	L.SEIQNIKSELKYVPRA.E[18]	Small muscular protein	[69-84]	PR, VP, RA, YVP, KY, EI, YV	VP, RA, EI, IQ, KS, KY, QN, YV	SE(2)	EL, LK
**17**	F.TSVVDTPEIIHAQQVKN.L	Nebulin	[6061-6077]	VK, EI, TP	VV, HA, TP, EI, IH, II, QQ, QV, SV, TS, VD, VK	II	-
**18**	E.EAPPPPAEVHEVHEEVH.E[19]	Troponin T, fast skeletal muscle	[22-38]	AP, EA, EV(3), PP(3), EAP, PPP(3)	PPPP, PP(3), AP, PA, AE,	EE	-

					EV(3), HE(2), VH(3)		
**19**	M.ARVREPVISAVEQTAQR.T[20]	Titin	[436-452]	VR, AR, VE, AV, RVR	EP, TA, VR, AV, PV, QT, VE, VI	-	-
**20**	H.SSTFDAGAGIALNDHFVKL.I[21]	Glyceraldehyde-3-phosphate dehydrogenase	[290-308]	VK, IA, GI, GA, AG, DA, KL, LN, TF, ST	GA, IA, AL, AG, GI, HF, LN, ND, TF, VK	-	VKL
**21**	W.ITKQEYDEAGPSIVHRK.C[22]	Actin, alpha skeletal muscle	[359-375]	GP, AG, EA, EY, AGP	GP, AG, EY, HR, PS, QE, RK, SI, TK, VH, YD	IV	-
**22**	W.ISKQEYDESGPSIVHRK.C[23]	POTE ankyrin domain family member	[1057-1073]	GP, SG, EY, SGP	GP, ES, EY, HR, PS, QE, RK, SI, SK, VH, YD	IV	-
**23**	N.FTSVVDTPEIIHAQQVKN.L[24]	Nebulin	[6060-6077]	VK, EI, TP	VV, HA, TP, EI, IH, II, QQ, QV, SV, TS, VD, VK	II	-
**24**	L.RVAPEEHPTLLTEAPLNPK.A[25]	Actin, aortic smooth muscle	[97-115]	VAP, LNP, PL, AP, EA, TE, LN, PTM HP, EAP	VA, AP(2), LL, HP, NP, PL, EH, LN, PK, PT, TE, TL	LL, EE	-
**25**	M.YKADLKDLSKKGYDLRTD.A[26]	Nebulin	[1052-1069]	GY, KG, YK, KA, LR	KA, AD, GY, KG, KK, SK, TD, YD, YK	-	KD, LK
**26**	G.VGKKAEAVATVVAAVDQARVR.E[27]	Titin	[696-716]	VAA, AA, VG, GK, EA, VR,	VA(2), KA, VV, VR, AA, AE, AT, AV(2), DQ,	-	-

				AR, KA, AV(2), RVR	KK, QA, TV, VD, VG		
**27**	M.WITKQEYDEAGPSIVHRK.C[28]	Actin, alpha skeletal muscle	[358-375]	GP, AG, EA, EY, AGP	GP, WI, AG, EY, HR, PS, QE, RK, SI, TK, VH, YD	IV	-
**28**	A.AVDMARVREPVISAVEQTAQR.T[29]	Titin	[432-452]	VR, AR, VE, AV, DM, RVR	MA, EP, TA, VR, AV, PV, QT, VD, VE, VI	-	-
**29**	C.DFPVEAGDKFIEALGHTWHDT.C[30]	LIM domain-binding protein	[668-689]	FP, AG, GH, LG, GD, EA(2), KF, EI, VE, DF	FP, AL, AG, GH, HD, HT, KF, PV, TW, VE, WH	-	TW
**30**	E.MEKANFTSVVDTPEIIHAQQVKN.L[31]	Nebulin	[6055-6077]	VK, NF, KA, EI, ME, EK, TP	KA, VV, HA, TP, EK, EI, IH, II, ME, NF, QQ, QV, SV, TS, VD, VK	II	-
**31**	R.APPPIAYNPLLSPFFPQAARTLPKAQ.S[32]	Synaptopodin 2-like protein	[807-832]	FP, LSP, AY, PL, IA, AP, AA, AR, KA, PP(2), PQ, IAY, PPP, YN	PP(2), KA, AP, LP, LL, SP, FP, IA, NP, AA, PL, AY, PF, PI, PK, PQ, QA, TL, YN	LL	AY
**32**	M.GVDPHQKVELGIDLLAYGAKAELPQYK.S[33]	Myozenin-1	[192-206]	YG, LAY, AY, LA, GI, GA, GV, LG, QK, YK, KA, VE, PQ, PH, LGI, AEL	KA, LA, LP, LL, GA, AE, AY, DP, GI, GV, KV, PH, PQ, QY, VD, VE, YG, LPQ	LL	AY, EL(2), PHQ

**33**	N.VKLNEHFLNTSDFLDTIKSNLDRALGRQ.-[34]	Isocitrate dehydrogenase [NADP], mitochondrial (Fragment)	[394-421]	VK, RA, GR, LG, KL, LN(2), DF	RA, FL(2), AL, DR, EH, HF, KS, LN(2), NE, NL, NT, TI, TS, VK,	-	SDF, VKL

[1] sign (.) indicate cutting points
[2] position in parental protein
[3] stimulating vasoactive substance release or glucose uptake stimulating peptide
[4] other activity: alpha-glucosidase inhibitor – EA; dipeptidyl peptidase III inhibitor – PE
[5] other activity: dipeptidyl peptidase III inhibitor – HF
[6] other activity: antiamnestic, antithrombotic, regulating the stomach mucosal membrane activity – PG; alpha-glucosidase inhibitor – PP; dipeptidyl peptidase III inhibitor – HF; renin inhibitor - LR
[7] other activity: CaMPDE inhibitor, renin inhibitor – KF
[8] other activity: CaMPDE inhibitor, renin inhibitor – KF
[9] other activity: neuropeptide – GQ
[10] other activity: antiamnestic, antithrombotic – GP; regulating ion flow – DY; regulating the stomach mucosal membrane activity – GP; bacterial permease ligand – KK;
[11] other activity: renin inhibitor – SF
[12] other activity: dipeptidyl peptidase III inhibitor – IH, PE
[13] other activity: antiamnestic, antithrombotic – GP; regulating ion flow – DY; regulating the stomach mucosal membrane activity – GP; bacterial permease ligand – KK
[14] the number in () indicates the number of identified peptides, if more than one
[15] other activity: dipeptidyl peptidase III inhibitor – YK, DA(2)
[16] other activity: dipeptidyl peptidase III inhibitor – KA; CaMPDE inhibitor and renin inhibitor - IR
[17] other activity: antiamnestic, antithrombotic – GP; regulating the stomach mucosal membrane activity – GP; alpha-glucosidase inhibitor – EA
[18] other activity: activating ubiquitin-mediated proteolysis – RA; dipeptidyl peptidase III inhibitor – PR
[19] other activity: dipeptidyl carboxypeptidase inhibitor – PPPA; alpha-glucosidase inhibitor – EA, PP(3)
[20] other activity: dipeptidyl peptidase III inhibitor – RV
[21] other activity: dipeptidyl peptidase III inhibitor – TF, DA, HF; renin inhibitor – TF
[22] other activity: antiamnestic, antithrombotic, regulating the stomach mucosal membrane activity – GP; alpha-glucosidase inhibitor - EA
[23] other activity: antiamnestic, antithrombotic, regulating the stomach mucosal membrane activity – GP
[24] other activity: dipeptidyl peptidase III inhibitor – IH, PE; renin inhibitor - FT
[25] other activity: dipeptidyl peptidase III inhibitor – RV, HP, PE; alpha-glucosidase inhibitor – EA
[26] other activity: dipeptidyl peptidase III inhibitor – LR, YK, KA; renin inhibitor - LR; bacterial permease ligand – KK
[27] other activity: dipeptidyl peptidase III inhibitor – RV, KA; bacterial permease ligand - KK; alpha-glucosidase inhibitor – EA
[28] other activity: antiamnestic, antithrombotic, regulating the stomach mucosal membrane activity – GP
[29] other activity: dipeptidyl peptidase III inhibitor – RV
[30] other activity: alpha-glucosidase inhibitor – EA(2); CaMPDE inhibitor and renin inhibitor – KF

[31]other activity: dipeptidyl peptidase III inhibitor – IH, PE, KA;   renin inhibitor – FT
[32]other activity: dipeptidyl peptidase III inhibitor – PF, KA; alpha-glucosidase inhibitor – PP(2)
[33]other activity: dipeptidyl peptidase III inhibitor – YK, LA, YG, KA; immunomodulating - YG; activating ubiquitin-mediated proteolysis – LA
[34]other activity: dipeptidyl peptidase III inhibitor – HF, FL(2); activating ubiquitin-mediated proteolysis - RA

Table 2. List of peptide sequences common for conventional and organic loins

	Sequence[1]	Protein	Position[2]	ACE inhibitor	DPP-IV inhibitor	Stimulating[3]	Antioxidant
**1**	Q.SPLPVIPHQK.D	LIM domain-binding protein 3	[98-107]	PLP, PL, IP, QK, PH	LP, IP, SK, PL, PH, PV, VI	-	PHQ
**2**	G.LPEALIPVQLL.W[4]	Sarcoplasmic/endoplasmic reticulum calcium ATPase 3 (Fragment)	[11-21]	IP, EA	LP, LL, IP, AL, LI, PV, QL, VQ	LI, LL	
**3**	K.LPPVVPVSKPGALR.R[6]	Telethonin	[139-153]	LPP,VP, GA, PG, KP, PP, LR	PP, LP, VP, VV, KP, GA, AL, PG, PV[5], SK, VS	-	KP
**4**	D.PPDIEFAKKVTNQ.V[7]	Nebulin	[259-271]	AKK, IE, PP	PP, FA, KK, KV, NQ, TN, VT	-	-
**5**	K.QLPPVVPVSKPGALR.R[8]	Telethonin	[139-153]	LPP,VP, GA, PG, KP, PP,LR	PP, LP,VP, VV, KP, GA, AL, PG, PV(2), QL, SK, VS	-	KP
**6**	P.PPPAEVHEVHEEVH.E[9]	Troponin T, fast skeletal muscle	[25-38]	EV(3), PP(2), PPP	PP(2), PA, AE, EV(3), HE(2), VH(3)	EE	KP
**7**	F.VAEPKESFVKGTVQS.R[10]	Myosin-1	[40-54]	VK, KG, GT, SF, KE	VA, EP, AE, ES, KE, KG, PK, QS, SF, TV, VK, VQ	-	-
**8**	A.RNATDIASQIKYKQ.S[11]	Nebulin	[6038-6051]	IA, KY, YK, IKY	IA, AS, AT, KY, NA, QI, RN, TD, YK	-	-

**9**	Q.MPKFDLGPLLSEPLV.L[12]	Myozenin-1	[240-254]	GPL, LGP, GP, PL(2), LG, KF, DLGP	GP, ML, LL, EP, PL(2), KF, LV, PK,	LV, LL, SE	-
**10**	E.TVFEKPKAGPAKSVFG.-[13]	Musculoskeletal embryonic nuclear protein 1	[67-82]	GPA, VF, GP, AG, FG, KA, KP, EK, AGP	GP, KA, PA, KP, GPA, EK, AG, KS, PK, SV, TV, VK(2)	-	KP
**11**	R.VREPVISAVEQTAQR.T	Titin	[438-452]	VR, VE, AV	EP, TA, VR, AV, PV, QT, VE, VI	-	-
**12**	S.VVDTPEIIHAQQVKN.L[14]	Nebulin	[6063-6077]	VK, EI, TP	VV, HA, TP, EI, IH, II, QQ, QV, VD, VK, IH	II	-
**13**	R.ARNATDIASQIKYKQ.S[15]	Nebulin	[6037-6051]	IA, KY, YK, AR, IKY	IA, AS, AT, KY, NA, QI, RN, TD, YK	-	-
**14**	T.IEPDAVHIKAAKDAYK.V[16]	Nebulin	[5525-5540]	AY, AA, DA(2), YK, KA, IE, IEP, AV	KA, EP, AA, AV, AY, HI, VH, YK	-	AY, KD
**15**	E.APPPPAEVHEVHEEVH.E[17]	Troponin T, fast skeletal muscle	[28-38]	AP, EV(3), PP(3), PPP(2)	PPPP, PP(3), AP, PA, AE, EV(3), HE(2), VH(3)	EE	-
**16**	Q.IRKETEKAFVPKVVIS.A[18]	Titin	[512-527]	IR, VPK, AF, VP, KA, TE, EK, KE, FVP	KA, VP, VV, EK, AF, ET, IR, KE, KV, PK, RK, TE, VI	-	IR
**17**	H.SSTFDAGAGIALNDHFVK.L[19]	Glyceraldehyde-3-phosphate dehydrogenase	[290-307]	VK, IA, GI, GA, AG(2), DA, LN, TF, ST	GA, AL., IA, AG(2); GI, HF, LN, ND, TF, VK,	-	-
**18**	C.TIEPDAVHIKAAKDAYK.V[20]	Nebulin	[5524-5540]	AY, AA, DA(2), YK KA, IE, IEP, AV	KA, EP, AA, AV, AY, HI, TI, VH, YK	-	AY, KD

**19**	F.TSVVDTPEIIHAQQVKN.L[21]	Nebulin	[6061-6077]	VK, EI, TP	VV, HA, TP, EI, IH, II, QQ, QV, SV, TS, VD, VK	II	-
**20**	H.AVPDTPQILLAKTVSNLV.S[22]	Nebulin	[5630-5647]	AVP, LA, VP, PQ, AV, TP, IL	LA, VP, LL, TP, AV, IL, KT, LV, NL,PQ, QI, TV, VS	LV, IL, LL	-
**21**	G.KGVLKAVEHINKTLGPAL.L[23]	Beta-enolase	[60-77]	GPA, LGP, GP, LKA, KG, GV, LG, NK, AK, VE, AV	GP, KA, PA, GPA, AL, AV, EH, GV, HI, IN, KG, KT, TL, VE, VL	VL	LK
**22**	L.KVSILAAIDEASKKLNAQ.-[24]	Apolipoprotein A-I	[248-265]	LAA, LA, AA, EA, KL, LN, AI, IL	LA, AA, AS, IL, KK, KV, LN, NA, SI, SK, VS	IL	-
**23**	E.EAPPPPAEVHEVHEEVH.E[25]	Troponin T, fast skeletal muscle	[22-38]	AP, EA, EV(3), PP(3), EAP, PPP(2),	PPPP, PP(3), AP, PA, AE, EV(3), HE(2), VH(3)	EE	-
**24**	M.ARVREPVISAVEQTAQR.T[26]	Titin	[436-452]	VR, AR, VE, AV, RVR	EP, TA, VR, AV, PV, QT, VE, VI	-	-
**25**	E.KAKDIEHAKKVSQQVSK.V[27]	Nebulin	[153-169]	AKK, KA, IE	KA, HA, EH, KK, KV, QQ, QV, SK, VS(2)	-	KD
**26**	T.IETRDGEVVSEATQQQH.E[28]	Desmin	[451-467]	GE, EA, DG, IE, EV, TQ	VV, AT, ET, EV, GE, QH, QQ(2),TQ, TR, VS	-	SE
**27**	S.SPVFPRAGFGTKGSSSSVTS.R[29]	Desmin	[32-51]	VF, FP, PR, RA, GF, AG, KG, FG, GS, GT	SP, FP, RA, AG, GF, KG, PV, SV, TK, TS, VF, VT	SSS(2)	-
**28**	R.VAPEEHPTLLTEAPLNPK.A[30]	Actin, alpha skeletal muscle	[98-115]	VAP, LNP, PL, AP(2), EA, TE, LN, PT, HP, EAP	VA, AP(2), LL, HP, NP, PL, EH, LN, LT, PK, PT, TE, TL	LL, EE	-

**29**	G.DWRKNIEEKSGMEGRK.K	Troponin T, fast skeletal muscle	[160-175]	GM, GR, SG, EG, IE, ME, EK	EK, WRK, WR, EG, KS, ME, RK	EE	-
**30**	H.SSTFDAGAGIALNDHFVKL.I[31]	Glyceraldehyde-3-phosphate dehydrogenase	[290-308]	VK, IA, GI, GA, AG(2), DA, KL, LN, TF, ST	GA, IA, AL, AG, GI, HF, LN, ND, TF, VK	-	VKL
**31**	T.RTTPTMKELQMMVEHH.L	Protein phosphatase 1 regulatory subunit	[76-91]	VE, LQ, PT, KE, TP, MM	TP, EH, HH, KE, MK,MM, MV, PT, TM, TT, VE	-	HH, EL, EHH, MM
**32**	W.ITKQEYDEAGPSIVHRK.C[32]	Actin, alpha skeletal muscle	[359-375]	GP, AG, EA, EY, AGP	GP, AG, EY, HR, PS, QE, RK, SI, TK, VH, YD	IV	-
**33**	K.MAPNIPLEMELPGVKIVH.A[33]	PDZ and LIM domain protein	[169-186]	LPG, PL, VK, IP, AP, GV, PG, ME, MAP	MA, AP, LP, IP, PL, GV, KI, ME, PG, PN, VH, VK	IV	EL
**34**	N.FTSVVDTPEIIHAQQVKN.L[34]	Nebilin	[6060-6077]	VK, EI, TP	VV, HA, TP, EI, IH, II, QQ, QV, SV, TS, VD, VK,	II	-
**35**	K.TIETRDGEVVSEATQQQH.E[35]	Desmin	[450-467]	GE, EA, DG, IE, EV, TQ	VV, AT, ET, EV, GE, QH, QQ, TI, TQ, TR, VS	SE	-
**36**	N.NEKEYKRDLELEVKGRG.N[36]	Nebulin	[6007-6023]	VK, KR, GR, KG, YK, EY, EV, EK, KE, RG	EK, EV, EY, KE, KG, KR, NE, RG, VK, YK	-	EL
**37**	G.DWRKNIEEKSGMEGRKK.M[37]	Troponin T, fast skeletal muscle	[160-176]	GM, GR, SG, EG, IE, ME, EK	EK, WRK, WR, EG, KK, KS, ME, RK(2)	EE	-
**38**	H.EQIRKETEKAFVPKVVIS.A[38]	Titin	[510-527]	IR, VPK, AF, VP, KA, TE, EK, KE, FVP	KA, VP, VV, EK, AF, ET, IR, KE, KV, PK, QI, RK, TE, VI		IR

**39**	L.RVAPEEHPTLLTEAPLNPK.A[39]	Actin, alpha skeletal muscle	[97-115]	VAP, LNP, PL, AP(2), EA, TE, LN, PT, HP, EAP	VA, AP(2), LL, HP, NP, PL, EH, LN, LT, PK, PT, TE, TL	LL, EE	-
**40**	M.YKADLKDLSKKGYDLRTD.A[40]	Nebulin	[1052-1067]	GY, KG, YK, KA, LR	KA, AD, GY, KG, KK, SK, TD, YD, YK	-	KD, LK
**41**	G.VGKKAEAVATVVAAVDQARVR.E[41]	Titin	[696-716]	VAA, AA, VG, GK, EA, VR, AR, KA, AV(2), RVR	VA(2), KA, VV, VR, AA, AE, AT, AV(2), DQ, KK, QA, TV, VD, VG	-	-
**42**	M.WITKQEYDEAGPSIVHRK.C[42]	Actin, alpha skeletal muscle	[358-375]	GP, AG, EA, EY, AGP	GP, WI, AG, EY, HR, PS, QE, RK, SI, TK, VH, YD	IV	
**43**	Q.SGKEYRKDYEESIKGRNL.T[43]	Nebulin	[5933-5950]	GR, KR, GK, SG, EY, KE, DY, YE	ES, EY, KE, KG, NL, RK, RN, SI, YE, YR	EE	KD
**44**	Q.SGKEYRKDYEESIKGRNLTG.L[44]	Nebulin	[5933-5952]	GR, KG, GK, SG, TG, EY, KE, DY, YE	ES, EY, KE, KG, LT, NL, RK, RN, SI, TG, YE, YR	EE	KD
**45**	C.HVDEKAKDIEHAKKVSQQVSK.V[45]	Nebulin	[149-169]	AKK, KA, IE, EK, KD	KA, HA, EK, EH, HV, KK, KV, QQ, QV, SK, VD, VS(2)	-	KD
**46**	F.GGAPSFPLGSPLSSPVFPRAGFGTK.G[46]	Desmin	[19-43]	VF, FP(2), PR, PLG, PL(2), AP, RA, GF, GA, AG, FG, GS, GT, GG, LG, SF	AP, SP(2), FP(2), GA, RA, PL(2), AG, GF, GG, PS, PV, SF, TK, VF	-	-
**47**	L.FVTNDAATILRELEVQHPAAKM.I[47]	T-complex protein 1 subunit theta	[64-85]	AA(2), DA, EV, HP, IL, LR	PA, HP, AA(2), AT, EV, IL, ND, QH, TI, TN, VQ, VT	IL	EL

**48**	L.KPRPPPPPPAPPKEDVKEKIFQ.L[48]	Titin	[11805-11826]	PR, VK, RP, AP, IF, PAP, PPK, KP, PP(6), EK, KE(2), PAPPK, RPP, FQ, PPP(4)	PPPP(4); PP(6), AP, PA, RP, KP, EK, FQ, KE(2), KI, PK, VK	-	KP
**49**	M.RFLGDEETVRKAMEAVAAQGKAK.-[49]	Phosphoglycerate mutase 2	[231-253]	RF, VAA, AA, GK, QG, LG, GD, EA, VR, KA(2), ME, AV	VA, KA, FL, VR, AA, AV, ET, ME, QG, RK, TV	EE	-
**50**	L.SSPVFPRAGFGTKGSSSSVTSRVYQ.V[50]	Desmin	[31-55]	VF, VY, FP, PR, RA, GF, AG, KG, FG, GS, GT, RVY	SP, FP, RA, AG, GF, KG, PV, SV, TK, TS, VF, VT, VY, YQ	SSS(2)	VY
**51**	E.MEKANFTSVVDTPEIIHAQQVKN.L[51]	Nebulin	[6055-6077]	VK, NF, KA, EI, ME, EK, TP	KA, VV, HA, TP, EK, EI, IH, II, ME, NF, QQ, QV, SV, TS, VD, VK	II	-
**52**	R.APPPIAYNPLLSPFFPQAARTLPK.A[52]	Synaptopodin 2-like protein	[807-830]	FP, LSP, AY, PL, IA, AP, AA, AR, PP(2), PQ, IAY, PPP, YN	PP(2), AP, LP, LL, SP, FP, IA, NP, AA, PL, AY, PF, PI, PK, PQ, QA, TL, YN	LL	AY
**53**	STGEALVQGLMGAAVTLKNLTGLNQRR[53]	Ubiquinone biosynthesis protein COQ9	[292-318]	AA, GA, GL(2), MG, GE, QG, TG(2), EA, RR, LN, AV, LVQ, ST	GA, AL, GL(2), AA, AV, GE, LM, LN, LT, LV, MG, NL, NQ, QG, RR, TG(2), TL, VQ, VT	LV	LK, GAA
**54**	SWYDNEFGYSNRVVDLMVHMASKE[54]	Glyceraldehyde-3-phosphate dehydrogenase	[314-321]	GY, FG, KE	MA VV, WY, AS, DN, GY, KE, LM, MV, NE, NR, SK, SW, VD, VH, YD, YS	-	WY
**55**	Y.MLTDRENQSILITGESGAGKTVNTKRVIQ.Y[55]	Myosin-6	[164-192]	KR, GA, AG, GK, GE, SC, TG, IL	GA, AG, DR, ES, GE, IL, IQ, KR, KT, LI, LT, ML, NQ,	IL, LI	-

					NT, QS, SI, TD, TG, TK, TV, VI, VN		
**56**	F.SSVLYKEDVSPGTAIGKTPEMMRVKQTQDH.I + Oxidation (M)[56]	Nebulin	[6150-6179]	LV, VSP, VK, IG, GK, GT, PG, YK, TQ, KE, AI, VLY, TP, MM	TP, SP, TA, KE, KT, MM, MR, PG, QD, QT, SV, TQ, VK, VL, VS, YK	VL	LY, MM
**57**	F.SKKLYTEDWEADKSLFYPYNDSPELRRVAQAQK.A[57]	Nebulin	[203-235]	LY, LF, FY, YP, EA, QK, KL, RR, TE, YN, LR	VA, SP, YP, SL, YPY, WE, YT, AD, KK, KS, ND, PY, QA, RR, SK, TE, YN	-	LY, EL, PEL
**58**	V.TPALLHVKYA.T[58]	Nebulin	[5956-5965]	VK, YA, KY, TP	PA, LL, TP, AL, HV, AY,	LL	LH, LHV
**59**	A.GIALNDHFVK.L[59]	Glyceraldehyde-3-phosphate dehydrogenase	[298-307]	VK, IA, GI, LN	IA, AL, GI, HF, LN, ND, VK		
**60**	E.VTPALLHVKYA.T[60]	Nebulin	[5955-5965]	VK, YA, KY, TP	PA, LL, TP, AL, HV, KY, LH, VK, VT, YA	LL	LH, LHV
**61**	E.EVIEVKVPAVH.T	Titin	[10153-10163]	VK, VP, IE, EV(2), AV	PA, VP, AV, EV(2), KV, VH, VI, VK	-	VKV
**62**	T.MEKAGAHLKGGAK.R[61]	Glyceraldehyde-3-phosphate dehydrogenase	[103-115]	GA(2), AG, HL, KG, GG, KA, AH, ME, EK	KA, GA(2), HL, EK, AG, AH, GG, KG, ME	-	HL, AH, GAH, LK
**63**	G.DAIPITAAKASRNIA.S[62]	Nebulin	[1313-1327]	AIP, IA, IP, AA, DA, KA, AJ	IPI, KA, IP, IA, TA, AA, AS, PI, RN	-	-
**64**	Q.SIVKYKEKYEKE.R[63]	Nebulin	[5898-5909]	VK, KY(2), YK, EK(2), KE(2), YE	EK(2), KE(2), KY(2), SI, VK, YE, YK	IV	-

**65**	G.DWRKNIEEKSGMEG.R	Troponin I, fast skeletal muscle	[160-173]	GM, SG, EG, IE, ME, EK	EK, WRK, WR, EG, KS, ME, RK	EE	-
**66**	G.SPLSSPVFPRAGFGTKG.S[64]	Desmin	[28-44]	VF, FP, PR, PL, RA, GF, AG, KG, FG, GT	SP(2), FP, RA, PL, AG, GF, KG, PV, TK, VF	-	-
**67**	M.GVNHEKYDNSLKIVSN.A	Glyceraldehyde-3-phosphate dehydrogenase	[136-147]	GV, KY, EK	EK, SL, DN, GV, HE, KI, KY, NH, VN, VS, YD	IV	LK
**68**	A.RVREPVISAVEQTAQR.T[65]	Titin	[437-452]	VR, VE, AV, RVR	EP, TA, VR, AV, PV, QT, VE, VI	-	-
**69**	Q.SLQDDPKLVHYMNVAK.I	Nebulin	[1104-1119]	HY, KL, LQ	VA, SL, DP, HY, LV, MN, NV, PK, QD, VH, YM	LV	-
**70**	G.DEETVRKAMEAVAAQGKAK.-[66]	Phosphoglycerate mutase	[235-253]	VAA, AA, GK, QG, EA, VR, KA(2), ME, AV	VA, KA(2), VR, AA, AV, ET, ME, QG, RK, TV	EE	-
**71**	L.MRIEEALGDKAVFAGRKF.R[67]	Beta-elonase	[411-428]	VF, AG, GR, LG, GD, EA, KF, KA, IE, AV	KA, FA, AL, AG, AV, KF, MR, RI, RK, VF	EE	-
**72**	G.FQSLQDDPKLVHYMNVAK.I	Nebulin	[1102-1119]	HY, KL, LQ, FQ	VA, SL, DP, FQ, HY, LV, MN, NV, PK, QD, QS, VH, YM	LV	-
**73**	S.HEQIRKETEKAFVPKVVIS.A[68]	Titin	[509-527]	IR, VPK, AF, VP, KA, TE, EK, KE	KA, VP, VV, EK, AF, ET, HE, IR, KE, KV, PK, QI, RK, TE, VI	-	FVP
**74**	L.ESKHPGDFGADAQGAMSKALEL.F[69]	Myoglobin	[117-137]	GA(2), FG, DA, QG, GD, PG, KA, HP, DF, DFG	KA, HP, GA(2), AL, AD, ES, KH, PG, QG, SK(2)	-	EL

**75**	E.DNPKSLKSGDAAIVEMVPGKPM.C[70]	Elongation factor 1-alpha 2	[389-910]	GKP, VP, AA, DA, GK, SG, GD, PG, KP, VE, AI	VP, KP, NP, SL, AA, DN, KS(2), MV, PG, PK, PM, VE	IV	LK, KP
**76**	G.SPLSSPVFPRAGFGTKGSSSSVTSRVYQ.V[71]	Desmin	[28-55]	VF, VY, FP, PR, PL, RA, GF, AG, KG, FG, GS, GT, RVY	SP(2), FP, RA, PL, AG, GF, KG, PV, SV, TK, TS, VF, VT, VY, YQ	SSS(2)	VY
**77**	D.DEEKKPIPGAKKLPGPAVNLSEIQNIK.S[72]	Small muscular protein	[49-75]	GPA, AKK, LPG, GP, IP, GA, PG, KL, KP, EI, EK, AV	GP, PA, LP, IP, KP, GPA, GA, EK, AV, EI, IQ, KK(2), NL, PG(2), PI, QN, VN	EE, SE	KP
**78**	G.TVPVGRVETGILRPGMVVTFAPVNITTEVKS.V[73]	Elongation factor 1-alpha 2	[261-292]	FAP, LRP, VK, RP, AP, VP, VG, GI, GM, GR, TG, PG, ITT, EV, VE, TE, TF, GILRP, IL, LR	FA, AP, VP, VV, RP, ET, EV, GI, IL, KS, MV, PG, PV(2), TE, TF, TG, TT, TV, VE, VG, VK, VN, VT	IL	-
**79**	S.SYRRTFGGAPSFPLGSPLSSPVFPRAGFGTKG.S[74]	Desmin	[13-44]	VF, FP(2), PR, PLG, PL(2), AP, RA, GF, GA, AG, KG, FG(2), GS, GT, GG, LG, SY, SF, RR, TF	AP, SP(2), FP(2), GA, RA, PL(2), AG, GF, GG, KG, PS, PV, RR, SF, SY, TF, TK, VF, YR	-	-
**80**	L.PGVKIVHA.Q[75]	PDZ and LIM domain protein 3	[181-188]	VK, GV, PG	HA, GV, KI, PG, VH, VK	IV	-
**81**	E.YDEAGPSIVH.R[76]	Actin, alpha skeletal muscle	[363-373]	GP, AG, EA, AGP	GP, AG, PS, SI, VH, YD	IV	-
**82**	F.DLGPLLSEPLVL.V[77]	Myozenin-1	[244-255]	LVL, GPL, LGP, GP, PL(2), LG, DLGP	GP, LL, EP, PL(2), LV, VL	VL, LV, LL, SE	-
**83**	K.ERDLRDVGDWRK.N[78]	Troponin I, fast skeletal muscle	[152-163]	VG, GD, LR	WRK, WR, RK, VG	-	-

**84**	I.PDLPEVKRVKETQKH.I[79]	Nebulin	[6196-6210]	DLP, VK(2), KR, QK, EV, TQ, KE	LP, ET, EV, KE, KH, KR, TQ, VK(2)	-	-
**85**	K.SEKERIEAQNKPFDAK.T[80]	Myosin-8	[22-37]	DA, EA, NK, KP, IE, EK, KE	KP, EK, KE, PF, QN, RI	SE	KP
**86**	GQKDSYVGDEAQSKRGILT[81]			KR, VG, GI, GQ, GD, EA, QK, SY, YV, IL, RG	GI, IL, KR, LT, QS, RG, SK, SY, VG, YV	IL	KD, YVGD
**87**	N.KPEDEADEWARRSSNLQS.R[82]	LIM domain-binding protein 3	[255-272]	EA, RR, AR, KP, LQ, EW, WA	KP, WA, AD, EW, NL, QS, RR	-	KP
**88**	Q.NKPEDEADEWARRSSNLQ.S[83]	LIM domain-binding protein 3	[254-271]	EA, NK, RR, AR, KP, LQ, EW, WA	KP, WA, AD, EW, NL, RR	-	KP
**89**	V.GDWRKNIEEKSGMEGRKK.M[84]	Troponin I, fast skeletal muscle	[159-176]	GM, GR, SG, GD, EG, IE, ME, EK	EK, WRK, WR, EG, KK, KS, ME, RK(2)	EE	-
**90**	Q.NKPEDEADEWARRSSNLQSR.S[85]	LIM domain-binding protein 3	[254-273]	EA, NK, RR, AR, KP, LQ, EW, WA	KP, WA, AD, EW, NL,QS, RR	-	KP
**91**	M.RIEEALGDKAVFAGRKFRNPKAK.-[86]	Beta-elonase	[412-434]	VF, FR, AG, GR, LG, GD, EA, KF, KA(2), IE, AV	KA(2), FA, NP, AL, AG, AV, FR, KF, PK, RI, RK, RN, VF	EE	-
**92**	M.ANETPDFMRARNATDIASQIKYKQ.S + Oxidation (M)[87]	Nebulin	[6028-6051]	IA, RA, KY, YK, AR, IKY, TP, DF	TP, IA, RA, AS, AT, ET, KY, MR, NA, NE, QI, RN, TD, YK	-	-
**93**	Q.SPLPVIPH.Q	LIM domain-binding protein 3	[98-105]	PLP, PL, IP, PH	LP, IP, SP, PL, PH, PV, VI	-	-

[1] sign (.) indicate cutting points
[2] position in parental protein
[3] Stimulating vasoactive substance release or glucose uptake stimulating peptide
[4]other activity: dipeptidyl peptidase III inhibitor- PE; alpha-glucosidase inhibitor – EA
[5] the number in () indicates the number of identified peptides, if more than one

[6]other activity: dipeptidyl peptidase III inhibitor - LP, antiamnestic (PEP inhibitor - LPPV, Prolyl endopeptidase inhibitor –PG; antithrombotic and peptide regulating the stomach mucosal membrane activity - PG; alpha-glucosidase inhibitor - PP; renin inhibitor – LR
[7] other activity: dipeptidyl peptidase III inhibitor - FA; bacterial permease ligand – KK; hypolipidemic   - EF; alpha-glucosidase inhibitor- PP; CaMPDE inhibitor and renin inhibitor – EF
[8] other activity: dipeptidyl peptidase III inhibitor- LR; antiamnestic (PEP inhibitor – LPPV, Prolyl endopeptidase inhibitor – PG; antithrombotic, peptide regulating the stomach mucosal membrane activity - PG; alpha-glucosidase inhibitor - PP; renin inhibitor - LR
[9] other activity: dipeptidyl carboxypeptidase inhibitor – PPPA; alpha-glucosidase inhibitor - PP(2)
[10] other activity: rennin inhibitor – SF;
[11] other activity: dipeptidyl peptidase III inhibitor – YK
[12] other activity: antiamnestic (prolyl endopeptidase inhibitor), antithrombotic   and regulating– GP; CaMPDE inhibitor and renin inhibitor – KF
[13] other activity: dipeptidyl peptidase III inhibitor - KA; antiamnestic (prolyl endopeptidase inhibitor), antithrombotic   and regulating– GP
[14] other activity: dipeptidyl peptidase III inhibitor- PE
[15] other activity: dipeptidyl peptidase III inhibitor- YK
[16] other activity: dipeptidyl peptidase III inhibitor- YK, DA(2)
[17] other activity: dipeptidyl carboxypeptidase inhibitor - PPPA, alpha-glucosidase inhibitor – PP(3)
[18] other activity: dipeptidyl peptidase III inhibitor- KA, CaMPDE inhibitor and renin inhibitor – IR
[19] other activity: dipeptidyl peptidase III inhibitor-TF, DA, HF, renin inhibitor – TF
[20] other activity: dipeptidyl peptidase III inhibitor - YK, DA(2), KA
[21] other activity: dipeptidyl peptidase III inhibitor – IH, PE
[22] other activity: dipeptidyl peptidase III inhibitor and activating ubiquitin-mediated proteolysis – LA
[23] other activity: dipeptidyl peptidase III inhibitor – KA; antiamnestic (prolyl endopeptidase inhibitor), antithrombotic and peptide regulating the stomach mucosal membrane activity – GP
[24] other activity: dipeptidyl peptidase III inhibitor and activating ubiquitin-mediated proteolysis – LA; bacterial permease ligand – KK; alpha-glucosidase inhibitor – EA
[25] other activity: alpha-glucosidase inhibitor – EA, PP(3); dipeptidyl carboxypeptidase inhibitor – PPPA
[26] other activity: dipeptidyl peptidase III inhibitor – RV
[27] other activity: dipeptidyl peptidase III inhibitor – KA; bacterial permease ligand – KK
[28] other activity: dipeptidyl peptidase III inhibitor – GE; alpha-glucosidase inhibitor – EA
[29] other activity: dipeptidyl peptidase III inhibitor – GF, PR; activating ubiquitin-mediated proteolysis – RA
[30] other activity: dipeptidyl peptidase III inhibitor - HP, PE;   alpha-glucosidase inhibitor – EA
[31] other activity: dipeptidyl peptidase III inhibitor – TF, DA, HF;   renin inhibitor – TF
[32] other activity: antiamnestic (prolyl endopeptidase inhibitor), antithrombotic and regulating the stomach mucosal membrane activity – GP; alpha-glucosidase inhibitor - EA
[33] other activity: antiamnestic (prolyl endopeptidase inhibitor) , antitrombotic and peptide regulating the stomach mucosal membrane activity - PG
[34] other activity: dipeptidyl peptidase III inhibitor – IH, PE; renin inhibitor – FT
[35] other activity: dipeptidyl peptidase III inhibitor – GE, alpha-glucosidase inhibitor - EA
[36] other activity: dipeptidyl peptidase III inhibitor - YK
[37] other activity: bacterial permease ligand - KK
[38] other activity: dipeptidyl peptidase III inhibitor – KA, CaMPDE inhibitor nad renin inhibitor – IR
[39] other activity: dipeptidyl peptidase III inhibitor – RV, HP, PE; alpha-glucosidase inhibitor - EA

[40] other activity: dipeptidyl peptidase III inhibitor – LR, YK, KA; bacterial permease ligand – KK
[41] other activity: dipeptidyl peptidase III inhibitor – RV, KA; bacterial permease ligand – KK; alpha-glucosidase inhibitor - EA
[42] other activity: antiamnestic (prolyl endopeptidase inhibitor) , antitrombotic and peptide regulating the stomach mucosal membrane activity - GP
[43] other activity: dipeptidyl peptidase III inhibitor and neuropeptide (Kyotorphin) – YR; peptide regulating ion flow - DY
[44] other activity: dipeptidyl peptidase III inhibitor and neuropeptide (Kyotorphin) - YR; peptide regulating ion flow   - DY
[45] other activity: dipeptidyl peptidase III inhibitor – KA; bacterial permease ligand – KK
[46] other activity: dipeptidyl peptidase III inhibitor – GF, PR; activating ubiquitin-mediated proteolysis - RA; opioid(gliadin 1 exorphin) – PLG; renin inhibitor – SF;
[47] other activity: dipeptidyl peptidase III inhibitor – LR, DA, HP; renin inhibitor – LR
[48] other activity: dipeptidyl peptidase III inhibitor - PR; antithrombotic - PPK; dipeptidyl carboxypeptidase inhibitor – PPPA, PPAP; alpha-glucosidase inhibitor - PP(6)
[49] other activity: dipeptidyl peptidase III inhibitor – RF, FL, KA(2); antithrombotic - DEE; alpha-glucosidase inhibitor - EA
[50] other activity: dipeptidyl peptidase III inhibitor – GF, PR, RV, VY; activating ubiquitin-mediated proteolysis – RA
[51] other activity: dipeptidyl peptidase III inhibitor – IH, PE, KA; renin inhibitor – FT
[52] other activity: dipeptidyl peptidase III inhibitor – PF; alpha-glucosidase inhibitor – PP(2)
[53] other activity: dipeptidyl peptidase III inhibitor – RR, GE; alpha-glucosidase inhibitor – EA
[54] other activity: dipeptidyl peptidase III inhibitor – RV; CaMPDE inhibitor and hypolipidemic – EF; renin inhibitor – EF, NR
[55] other activity: dipeptidyl peptidase III inhibitor – GE, RV
[56] other activity: dipeptidyl peptidase III inhibitor – GE, RV; renin inhibitor – LY; peptide regulating the stomach mucosal membrane activity - PG
[57] other activity: dipeptidyl peptidase III inhibitor – LR, RR, RV, PE; renin inhibitor – LR, LY; bacterial permease ligand – KK; alpha-glucosidase inhibitor - YP, YPY, EA
[58] other activity; renin inhibitor – YA
[59] other activity: dipeptidyl peptidase III inhibitor – HF
[60] other activity; renin inhibitor – YA
[61] other activity: dipeptidyl peptidase III inhibitor – HL, KA
[62] other activity: dipeptidyl peptidase III inhibitor – DA, KA
[63] other activity: dipeptidyl peptidase III inhibitor – YK
[64] other activity: dipeptidyl peptidase III inhibitor – GF, PR; activating ubiquitin-mediated proteolysis - RA
[65] other activity: dipeptidyl peptidase III inhibitor – RV
[66] other activity: dipeptidyl peptidase III inhibitor – KA(2), antithrombotic - DEE, alpha-glucosidase inhibitor - EA
[67] other activity: dipeptidyl peptidase III inhibitor – KA, MR, FA; CaMPDE inhibitor and renin inhibitor – KF; alpha-glucosidase inhibitor - EA
[68] other activity: dipeptidyl peptidase III inhibitor – KA; CaMPDE inhibitor and renin inhibitor – IR
[69] other activity: dipeptidyl peptidase III inhibitor – DA, HP, KA; antiamnestic (prolyl endopeptidase inhibitor), antithrombotic and peptide regulating the stomach mucosal membrane activity – PG
[70] other activity: dipeptidyl peptidase III inhibitor – DA; antiamnestic (prolyl endopeptidase inhibitor), antithrombotic and peptide regulating the stomach mucosal membrane activity – PG
[71] other activity: dipeptidyl peptidase III inhibitor – GF, PR, RV, VY; activating ubiquitin-mediated proteolysis - RA
[72] other activity: antiamnestic (prolyl endopeptidase inhibitor) – PGP, PG(2), GP; antithrombotic – GP, PGP, PG(2), DEE; peptide regulating the stomach mucosal membrane activity – GP, PG(2), PGP; bacterial permease ligand – KK; inhibitor of insulin secretion and chemotactic – PGP;
[73] other activity: dipeptidyl peptidase III inhibitor – LR, TF, RV, FA; antiamnestic (prolyl endopeptidase inhibitor), antithrombotic and peptide regulating the stomach mucosal membrane activity – PG; renin inhibitor - LR, TF

[74] other activity: dipeptidyl peptidase III inhibitor – YR, RR, TF, GF, PR; activating ubiquitin-mediated proteolysis - RA, opioid (gliadin 1 exorphin) – PLG; neuropeptide (Kyotorphin) – YR; renin inhibitor – SF, TF
[75] other activity: antiamnestic (prolyl endopeptidase inhibitor), antithrombotic and peptide regulating the stomach mucosal membrane activity – GP
[76] other activity: antiamnestic (prolyl endopeptidase inhibitor), antithrombotic and peptide regulating the stomach mucosal membrane activity – GP
[77] other activity: antiamnestic (prolyl endopeptidase inhibitor), antithrombotic and peptide regulating the stomach mucosal membrane activity – GP
[78] other activity: dipeptidyl peptidase III inhibitor and renin inhibitor – LR
[79] other activity: dipeptidyl peptidase III inhibitor and renin inhibitor – RV, PE
[80] other activity: dipeptidyl peptidase III inhibitor and renin inhibitor – DA, PF; alpha-glucosidase inhibitor - EA
[81] other activity: neuropeptide - GQ; alpha-glucosidase inhibitor - EA
[82] other activity: dipeptidyl peptidase III inhibitor – RR, PE; peptide activating ubiquitin-mediated proteolysis – WA; alpha-glucosidase inhibitor - EA
[83] other activity: dipeptidyl peptidase III inhibitor – RR, PE; activating ubiquitin-mediated proteolysis - WA, alpha-glucosidase inhibitor - EA
[84] other activity: bacterial permease ligand – KK
[85] other activity: dipeptidyl peptidase III inhibitor – RR, PE; activating ubiquitin-mediated proteolysis – WA; alpha-glucosidase inhibitor – EA
[86] other activity: dipeptidyl peptidase III inhibitor – FA, FR, KA(2); CaMPDE inhibitor and renin inhibitor – KF; alpha-glucosidase inhibitor - EA

Table 3. List of peptide sequences common for conventional and organic shoulders

	Sequence[1]	Protein	Position[2]	ACE inhibitor	DPP-IV inhibitor	Stimulating[3]	Antioxidant
**1**	L.EMELPGVKIVHA.Q[4]	PDZ and LIM domain protein	[177-188]	LPG, VK, GV, PG, ME	LP, HA, GV, KI, ME, PG, VH, VK	IV	EL
**2**	R.VREPVISAVEQTAQR.T	Titin	[438-452]	VR, VE, AV	EP, TA, VR, AV, PV, QT, VE, VI	-	-
**3**	T.IEPDAVHIKAAKDAYK.V[6]	Neulin	[5525-5540]	AY, AA, DA(2)[5], YK, KA, IE, IEP, AV	KA, EP, AA, AV, AY, HI, VH, YK	-	AY, KD
**4**	E.APPPPAEVHEVHEEVH.E[7]	Troponin T, fast skeletal muscle	[23-38]	AP, EV(3), PP(3), PPP(2)	PPPP, PP(3), AP, PA, AE, EV(3), HE(2), VH(3)	EE	-
**5**	E.EAPPPPAEVHEVHEEVH.E[8]	Troponin T, fast skeletal muscle	[22-38]	AP, EA, EV(3), PP(3), EAP, PPP(2)	PPPP, PP(3), AP, PA, AE, EV(3), HE(2), VH(3)	EE	-
**6**	E.KAKDIEHAKKVSQQVSK.V[9]	Nebulin	[153-169]	AKK, KA, IE	KA, HA, EH, KK, KV, QQ, QV, SK, VS(2)	-	KD
**7**	T.IETRDGEVVSEATQQQH.E[10]	Desmin	[451-467]	GE, EA, DG, IE, EV, TQ	VV, AT, ET, EV, GE, QH, QQ(2), TQ, TR, VS	SE	-
**8**	R.VAPEEHPTLLTEAPLNPK.A[11]	Actin, aortic smooth muscle	[97-115]	VAP, LNP, PL, AP(2), EA, TE, LN, PT, HP, EAP	VA, AP(2), LL, HP, NP, PL, EH, LN, LT, PK, PT, TE, TL	LL, EE	-
**9**	W.ITKQEYDEAGPSIVHRK.C[12]	Actin, alpha skeletal muscle	[359-375]	GP, AG, EA, EY, AGP	GP, AG, EY, HR, PS, QE, RK, SI, TK, VH, YD	IV	-

**10**	M.WITKQEYDEAGPSIVHRK.C[13]	Actin, alpha skeletal muscle	[358-375]	GP, AG, EA, EY, AGP	GP, WI, AG, EY, HR, PS, QE, RK, SI, TK, VH, YD	IV	-
**11**	A.MSGMEGRKKMFDAAKSPTSQ.-[14]	Troponin I, slow skeletal muscle	[165-184]	MF, AA, GM, GR, DA, SG, EG, PT, ME	SP, AA, EG, KK, KS, ME, MF, PTM RK, TS	-	-
**12**	M.RFLGDEETVRKAMEAVAAQGKAK.-[15]	Phosphoglycerate mutase 2	[231-253]	RF, VAA, AA, GK, QG, LG, GD, EA, VR, KA(2), ME, AV	VA, KA(2), KL, VR, AA, AV, ET, ME, QG, RK, TV	EE	-
**13**	H.YTTVADRPDIKKATQAAKQ.A[16]	Nebulin	[5736-5754]	RP, AA, KA, TQ	VA, KA, RP, AA, YT, AD, AT, DR, KK, QA, TQ, TT, TV	-	IKK
**14**	Y.MVGPIEEAVAKADKLAEEHS.-[17]	ATP synthase subunit beta	[509-528]	GP, LA, VG, EA, KL, KA, IE, AV, VGP	GP, VA, KA, LA, AD, AE, AV, EH, HS, MV, PI, VG	EE(2)	-
**15**	R.DVGDWRKNIEEKSGMEGRKK.M[18]	Troponin T, fast skeletal muscle	[157-176]	VG, GM, GR, SG, DG, EG, IE, ME, EK	EK, WRK, WR, EG, KK, KS, ME, RK(2), VG	EE	-

[1] sign (.) indicate cutting points
[2] position in parental protein
[3] stimulating vasoactive substance release or glucose uptake stimulating peptide
[4] other activity: antiamnestic, antitrombotic, regulating the stomach mucosal membrane activity - PG
[5] the number in () indicates the number of identified peptides, if more than one
[6] other activity: dipeptidyl peptidase III inhibitor – YK, DA(2), KA
[7] other activity: dipeptidyl carboxypeptidase inhibitor – PPPA; alpha-glucosidase inhibitor – PP(3)
[8] other activity: dipeptidyl carboxypeptidase inhibitor – PPPA; alpha-glucosidase inhibitor – PP(3)
[9] other activity: dipeptidyl peptidase III inhibitor – KA; bacterial permease ligand - KK
[10] other activity: dipeptidyl peptidase III inhibitor – GE; alpha-glucosidase inhibitor – EA
[11] other activity: dipeptidyl peptidase III inhibitor – HP, PE; alpha-glucosidase inhibitor – EA
[12] other activity: antiamnestic, antitrombotic, regulating the stomach mucosal membrane activity – GP; alpha-glucosidase inhibitor – EA
[13] other activity: antiamnestic, antitrombotic, regulating the stomach mucosal membrane activity – GP; alpha-glucosidase inhibitor – EA
[14] other activity: dipeptidyl peptidase III inhibitor – DA; bacterial permease ligand – KK

[15] other activity: dipeptidyl peptidase III inhibitor – RF, FL, KA(2); antithrombotic - DEE
[16] other activity: dipeptidyl peptidase III inhibitor – KA; bacterial permease ligand - KK
[17] other activity: dipeptidyl peptidase III inhibitor – LA, KA; antiamnestic, antitrombotic, regulating the stomach mucosal membrane activity – GP; activating ubiquitin-mediated proteolysis - LA
18 other activity: bacterial permease ligand - KK
